# Epidemiology of human and animal leptospirosis in Kenya: A systematic review and meta-analysis of disease occurrence, serogroup diversity and risk factors

**DOI:** 10.1371/journal.pntd.0012527

**Published:** 2024-09-27

**Authors:** Martin Wainaina, Joseph Wasonga, Elizabeth Anne Jessie Cook

**Affiliations:** 1 Department of Biological Safety, German Federal Institute for Risk Assessment, Berlin, Germany; 2 Animal and Human Health Department, International Livestock Research Institute, Nairobi, Kenya; University of Minnesota, UNITED STATES OF AMERICA

## Abstract

**Background:**

Leptospirosis is a priority zoonotic disease in Kenya, but an in-depth review of its presence in humans, animals and the environment is lacking. Therefore, we conducted this systematic review and meta-analysis to understand the epidemiological situation to date.

**Methodology:**

We searched for literature in African journals online, AGRIS, Embase, the *Leptospira* WOAH reference laboratory library, ProMED-mail, PubMed, Scopus, Web of Science, and the institutional repositories of 33 academic institutions and included 66 publications on leptospirosis in Kenya which spanned from 1951 to 2022. The review was registered on the International Platform of Registered Systematic Review and Meta-analysis Protocols (INPLASY).

**Findings:**

Most investigations were done in rural and urban areas in western, southern, central, and coastal areas in Kenya and the largely pastoral eastern and northern areas were under-represented. A wide host range of domestic animals and wildlife was revealed, and occupational exposure was an important risk factor for humans. The microscopic agglutination test (MAT) was the most frequent test, particularly common in studies conducted during the 1980s and 1990s. However, varying MAT panels and cut-off titres were observed. The overall seroprevalence in cattle was 28.2% (95% confidence intervals [CI]: 12.0–53.0; heterogeneity: *I*^2^ = 96.7%, τ^2^ = 1.4), and 11.0% in goats (95% CI: 5.4–21.2; heterogeneity: *I*^2^ = 78.8%, τ^2^ = 0.4). Molecular tests were seldom used to determine species and illustrate strain diversity. There was a lack of awareness of leptospirosis among farmers and health practitioners.

**Conclusion:**

The widespread presence of leptospires and inadequate diagnostic capacity demonstrate that leptospirosis is a common but underreported disease in Kenya. Raising awareness and boosting the country’s diagnostic capacity is crucial to timely detection and disease control.

## Author summary

Leptospirosis is a prevalent but understudied zoonotic disease in Kenya. A comprehensive review of the presence of the disease in various hosts is therefore needed to catalogue the research efforts and inspire future scientific inquiry on this neglected tropical disease. We therefore undertook this review of published literature in leading databases and institutional repositories of relevant Kenyan higher institutions of learning. Literature spanning more than 70 years was identified and study areas showed a regional bias towards central, western, southern and coastal regions. The arid and semi-arid areas in the northern and eastern regions with large animal and wildlife populations that could facilitate leptospiral transmission were under-represented, and the inclusion of these pastoral regions could give a better picture of the country’s leptospirosis risk profile. Summary estimates in cattle and goats revealed considerable pooled seroprevalence of leptospirosis and the need for control in animal hosts. A low awareness among farmers, and animal and public health practitioners was found, demonstrating the need for education initiatives that can facilitate at-risk populations such as farmers. Additionally, the country lacks the capacity for MAT testing, and recent efforts to culture for leptospires are non-existent. Understanding the circulating serovars and strain diversity is crucial to the effective control of leptospirosis. Investments in diagnostics for peripheral and central laboratories are vital for public health response.

## Introduction

Leptospirosis is a common but neglected zoonotic disease in the world [1]. It is caused by bacteria of the genus *Leptospira* which are finely coiled, obligate aerobic, Gram-negative, catalase-producing, flagellated spirochetes [2]. The genus includes more than 250 serovars and species are currently classified based on whole genome sequencing (WGS) into the P clade and S clade which consist of subclades P1 (formerly pathogenic), P2 (formerly intermediates), S1 (formerly saprophytes) and S2 (a new subclade) [3].

*Leptospira* can infect a wide range of mammalian hosts which can act as reservoirs of the bacteria, but rodents are the most important. Humans primarily acquire leptospirosis either directly via contact with an infected animal or indirectly when in contact with soil or water contaminated with urine from an infected animal [4]. After infection, the bacteria colonise the renal tubules where they multiply and are eventually shed in urine. Exposure to the bacteria can happen occupationally (e.g., slaughterhouse work), recreationally (e.g., through water sports) or after extreme weather events such as heavy rainfall and flooding [4]. Areas with inadequate housing and poor sanitation such as informal settlements have an increased risk of exposure [4]. Human-to-human transmission is rare [5].

Human leptospirosis can initially present as an acute febrile illness which may resolve after a short period. Some patients can present with the severe late-phase stages of the disease which tend to be fulminant, Weil’s disease and leptospirosis-associated pulmonary haemorrhage syndrome being examples having high case fatality rates [6]. Animal leptospirosis is characterised by decreased productivity and reproductive losses such as abortions and stillbirths [7]. Leptospires tend to exhibit host-specificity due to the evolution of their virulence genes to adapt to their preferred hosts [8, 9]. An example of this is the Malagasy *Leptospira* which show specificity towards tenrecs and other small mammals of Madagascar [10].

Leptospirosis is an important cause of febrile illnesses in Africa [11] and a zoonotic disease prioritised for control in Kenya [12]. A recent outbreak of leptospirosis in Tanzania that affected 20 people and caused three fatalities indicates that the disease may be re-emerging in the region [13]. An in-depth review of the range of hosts, risk factors, diagnostic methods used, prevalent leptospiral serovars and prevalence estimates in various hosts will improve knowledge of this neglected zoonosis in the country. This is critical in control and prevention strategies. Therefore, we conducted this systematic review of leptospirosis in humans and animals in Kenya to answer these questions, reveal key knowledge gaps to inspire future research and inform control strategies.

## Methods

### Literature search

We searched for literature according to the Preferred Reporting Items for Systematic Reviews and Meta-Analyses (PRISMA) guidelines [14] in African journals online, the AGRIS (FAO) database, Embase, ProMED-mail, PubMed, Scopus, Web of Science, and the institutional repositories of 33 Kenyan universities and colleges. The review was registered on the International Platform of Registered Systematic Review and Meta-analysis Protocols (INPLASY) under registration number INPLASY202470097. We used the search terms “leptospirosis”, “leptospira”, “leptospir*”, “Weil disease”, “Weil’s disease”, “Weils disease”, “Spirochaetal jaundice”, “human”, “wildlife”, “domestic”, “rodent”, “ruminant”, “cattle/bovine”, “camel/dromedary”, “sheep/ovine”, “goat/caprine”, “prevalence”, “incidence”, “prevention”, “control”, “risk” and “Kenya”. The search terms were combined using different Boolean operators and the terms were modified to fit the needs of the various databases as presented in S1 Data. We set no time limit and allowed all languages. The searches were conducted on 14 February 2023. Lastly, we checked for relevant literature on isolates originating from Kenya on the website of the World Organisation for Animal Health (WOAH) reference laboratory for leptospirosis [15].

### Inclusion and exclusion criteria

We imported references and their abstracts into EndNote software 21 (Thomson Reuters, Philadelphia, PA, USA) and removed duplicate records. We screened the remaining records by title and abstract for eligibility. Studies based on animal and human populations in/originally from Kenya investigating any aspect (both qualitative and quantitative research) of leptospirosis were retained. We excluded records not focussed on leptospirosis, those investigating non-Kenyan populations, and those focussing on travellers. We allowed burden of disease estimations, case reports, case series, cohort, cross-sectional, diagnostic evaluations, outbreak investigations, post-mortem investigations, qualitative studies and study proposals. Grey literature was also permitted (conference proceedings, abstracts, reports and theses). We excluded books and book sections and scanned the reference sections of relevant reviews for articles not captured in our database searches.

### Data extraction

Two authors (MW and JW) screened the articles for inclusion and extracted the data and disagreements on the inclusion criteria were settled in consultation with the third author (EAJC). The DeepL translation tool was used for non-English and non-German literature [16]. Data on the study location, study hosts (animal/human/both), study type, study period, human population settings (community or hospital), animal species, diagnostic test applied, samples tested, number tested and number positive, leptospiral species/serovars/serogroups identified, culture and/or PCR technique, and risk factors for exposure were extracted when available. Clinical signs and symptoms in humans were also obtained from leptospirosis cases confirmed by culture or antibody titre rise.

### Statistical analysis

The data were imported into R version 4.3.2 where summary statistics were computed and plots and maps were generated to represent the spatial and temporal distribution of publications. Additionally, the microscopic agglutination test (MAT) panel diversity was plotted and the serogroups identified in various hosts by culture and serological methods were illustrated using an alluvial diagram made by the *ggalluvial* package [17]. Lastly, the hosts were categorised into three epidemiological compartments (human, domestic animals and wildlife) and the serogroups determined by MAT titres ≥1:100 (presence/absence data) were used to calculate the serogroup diversity (richness and evenness) by computing the Shannon, Simpsons, and Inverse Simpson indexes using the *vegan* package [18]. The Kruskal-Wallis test was applied to each of the three diversity indexes to assess statistically significant differences across the three compartments.

### Meta-analysis

Due to the paucity of data and diverse diagnostic methods and cut-offs utilised to determine positive cases, we did not estimate the summary effects in sheep and humans (considering community and hospital subpopulations). However, cross-sectional studies published in peer-reviewed journal articles that reported the prevalence in cattle and goats as tested from blood, serum or kidney samples using all diagnostic tests (MAT titres ≥1:100) were utilised in the meta-analysis to minimise the risk of bias. A random effects model was fitted on untransformed proportions. The *metaprop* command in the *meta* package [19] was used to pool studies using the logit transformed proportions (PLO) option. Study heterogeneity (Tau square or τ^2^) was estimated using the restricted maximum likelihood (REML) method and a Hartung-Knapp adjustment was applied. Higgins’ *I*^2^ statistic that quantifies study heterogeneity was reported as well. The results were visualised on forest plots with the diagnostic tests utilised as sub-groups and studies were arranged according to the publication year to illustrate any trends in prevalence with time.

## Results

**Fig 1:**
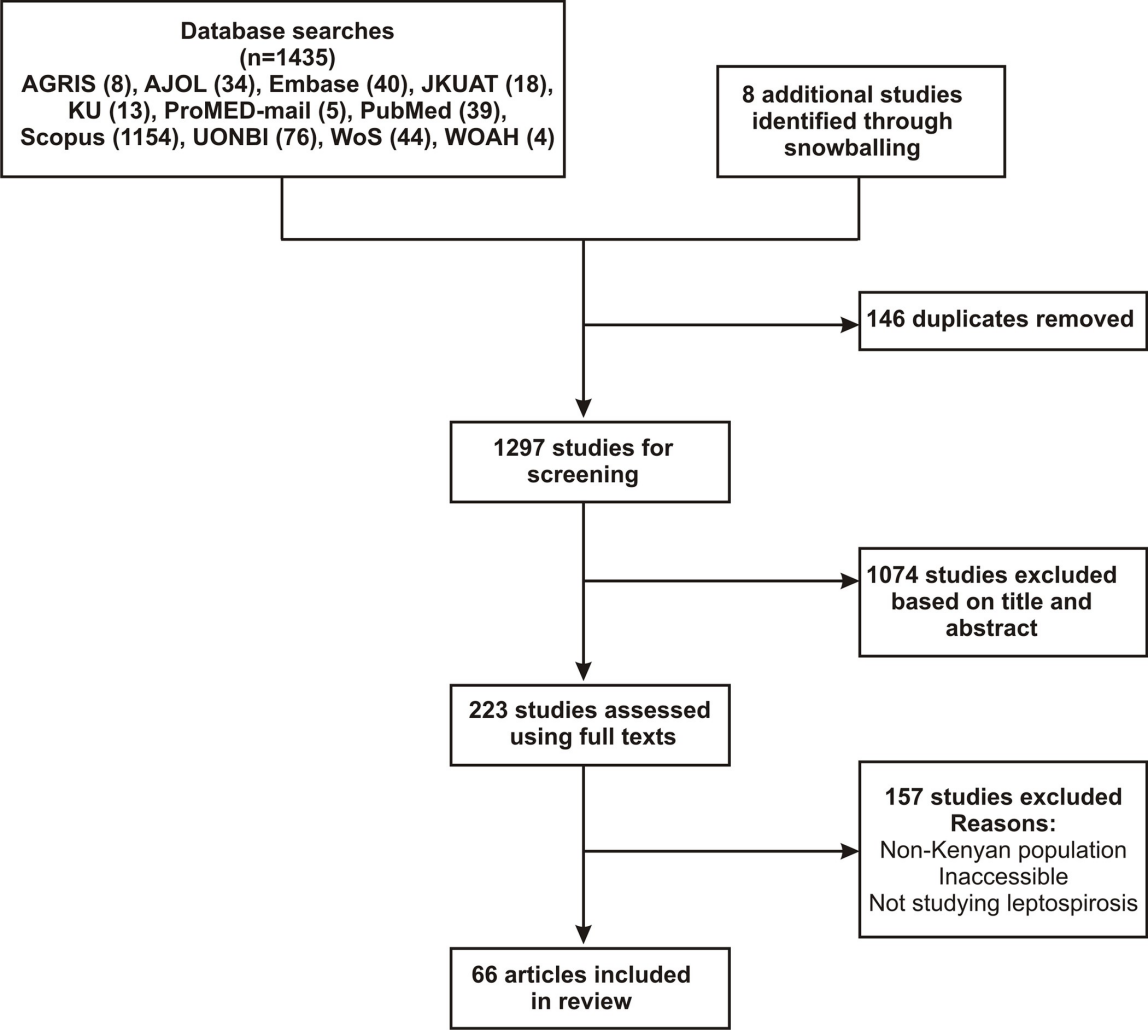
A PRISMA flow chart outlining the systematic review process to select relevant publications reporting on any aspect of leptospirosis in Kenya.

We initially identified 1,435 records from our database search and 66 were finally included in the qualitative and quantitative analyses. A summary of the study selection process is presented in Fig 1. Articles that were finally included were in English, Dutch or German.

**Fig 2:**
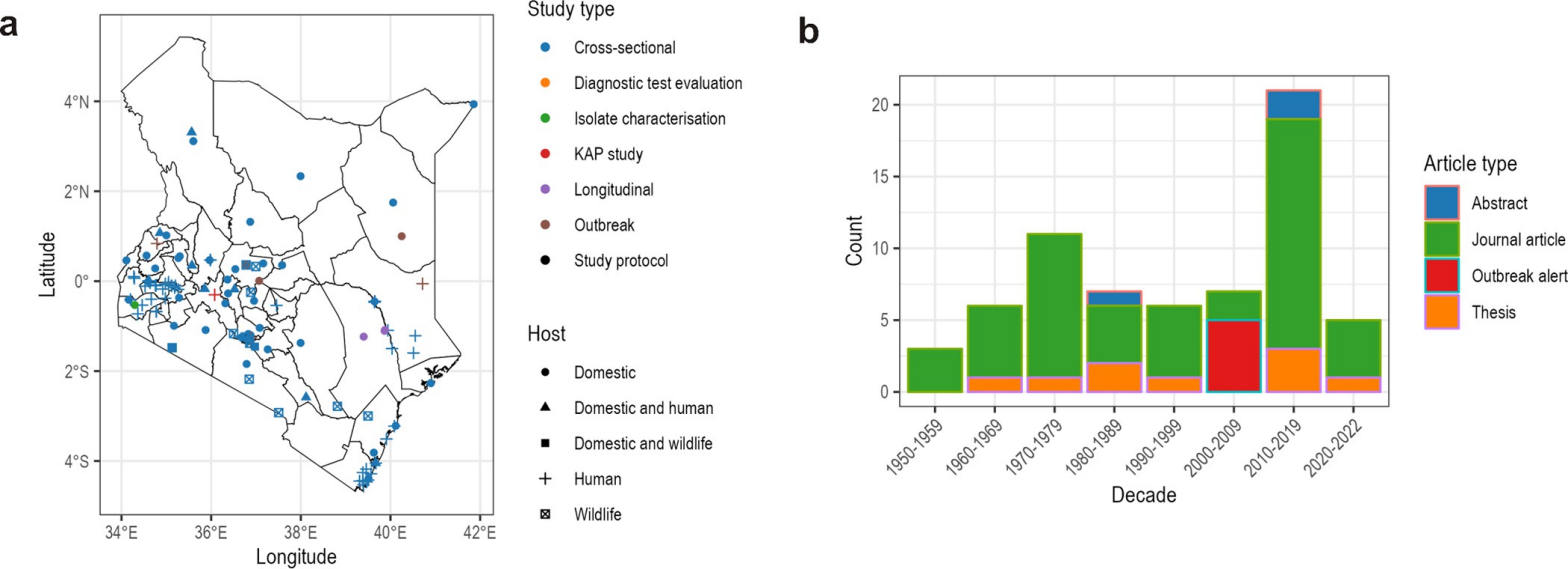
A summary of the literature on leptospirosis in Kenya. **a** Study sites of the included records investigating leptospires in Kenya presented by study types for various hosts. **b** Various article types investigating leptospires from 1951 to 2022 (geoBoundaries, https://www.geoboundaries.org/countryDownloads.html).

The included publications were widely distributed across Kenya (Fig 2), spanning from 1951 to 2022. There was an initial increase in the publication numbers until the 1970s, followed by a subsequent decline until 2010 when a sharp spike in the publication numbers was observed. Additionally, most studies focused on the central, coastal, southern and western regions of the country, and the expansive arid and semi-arid lands in the eastern and northern regions received less attention.

### Study characteristics

Journal articles were common (n = 49), and a few theses (n = 9), outbreak alerts on ProMED mail (n = 5) and abstracts (n = 3) were found. Cross-sectional studies formed the majority of the included publications (n = 37). Other types of reports included outbreak alerts and investigations (n = 11), diagnostic test evaluations (n = 4), isolate characterisations (n = 4), investigations on the knowledge, attitudes and practices (KAP) [n = 3], post-mortem characterisations of leptospirosis cases [pathology studies] (n = 2), longitudinal/prospective cohort (n = 1), the protocol of a proposed study [study protocol] (n = 1), and estimation of the burden of disease in Kenya via disability-adjusted life years (DALY) and incidence rates (n = 2). Studies investigating humans (n = 33) and domestic animals (n = 22) were several and a few investigated wildlife (n = 6), both humans and domestic animals (n = 2), humans, domestic animals and wildlife (n = 1), and domestic animals and wildlife (n = 1).

Of the studies that involved humans (n = 37), the majority examined participants in community settings (n = 17) and outpatients in hospital settings (n = 15), and a few recruited both hospital and community participants (n = 5). A summary of the characteristics of studies on animals and human hosts is presented in S1 Table.

### Diagnostic methods

**Fig 3:**
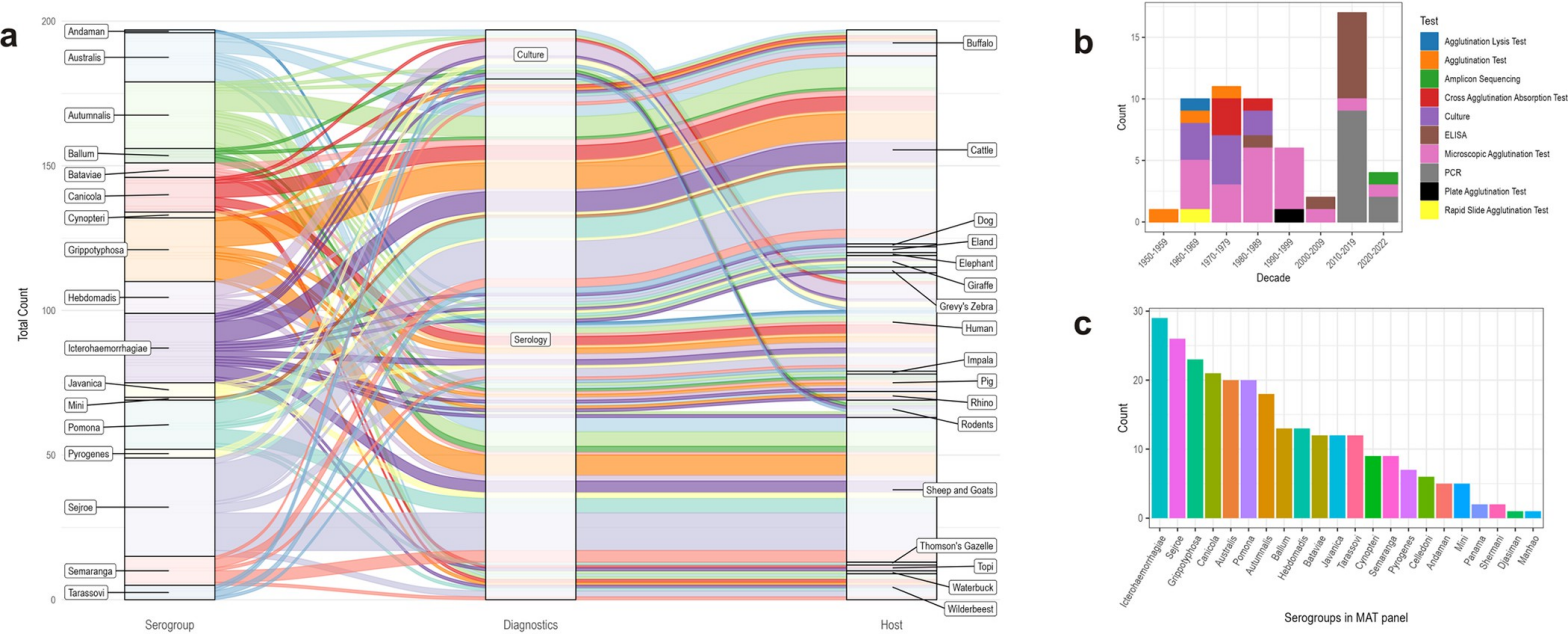
**a** Flow diagram illustrating the distribution of serogroups identified by culture and serological methods in various hosts in the country. Each stream presented in a unique colour and width represents the interconnectedness and number of cases in each category. **b** A distribution of the diagnostic tests applied to determine and characterise leptospires in the included studies over time. **c** Bar plot showing the count of serogroups represented in microscopic agglutination test (MAT) panels from included studies.

Serological testing was the most prominently found diagnostic method used in the study, applied in 34 studies. The serological gold standard diagnostic test, MAT, was the most commonly utilised to determine serovars (18/30). MAT panels used varied in composition and set different cut-offs of 1:30, 1:40, 1:50, 1:100, 1:200, and 1:3000 (S1 Table). Notably, two of these studies were from the 1960s, three from the 1970s, five from the 1980s, five from the 1990s, one from the 2000s and the remaining two from 2019 and 2022 (Fig 3).

Commercially available ELISA tests were used in a few studies (n = 8) [20–27] which all collectively identified serogroups Australis, Canicola, Celledoni, Djasiman, Grippotyphosa, Hebdomadis, Icterohaemorrhagiae, Mini, Pomona, Sejroe, and Tarassovi.

Eleven studies investigated leptospires using molecular tests, targeting the ribosomal 16S rRNA gene (*rrs*) via direct PCR [28–30] and amplicon sequencing [31], *lipL32* [32–34], *secY* [24,35,36], and *flaB* [33] genes. One study (conference abstract) lacked information on the PCR target [37]. All studies utilising PCR were conducted between the years 2011 and 2022 (Fig 3).

Culture was used in seven studies to cultivate leptospires from whole blood and kidney tissues using Cox’s [38], Ellinghausen–McCullough–Johnson–Harris (EMJH) [39], Fletcher’s [38,40–42] and Korthof’s media [40,43,44] with animal inoculation being used in some studies [38,40,43,44]. We found no recent studies utilising culture, with the last one being published in 1987 (Fig 3). Lastly, the first descriptions of the serovars (reference strain, serogroup) kanana (Kanana, Tarassovi) [45], lambwe (Lambwe, Autumnalis) [45], kenya (Njenga, Ballum) [45], kwale (Julu, Pyrogenes) [46], ramisi (Musa, Australis) [47], and nyanza (Kibos, Hebdomadis) [48] were found as determined by the cross-agglutination absorption test (CAAT).

### Prevalence distribution

**Fig 4:**
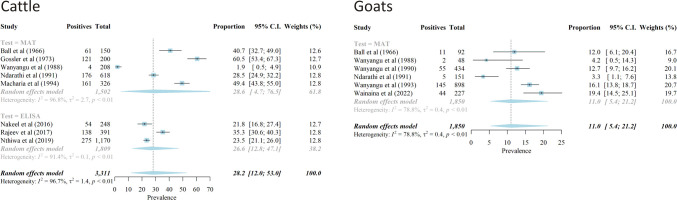
Forest plots of the meta-analysis of studies reporting seroprevalence in cattle and goats with subgroup analyses based on the diagnostic tests applied.

Several studies surveyed leptospires using serological and molecular tests in domestic animals which included cattle (n = 16) [21–23,36,38,39,43,44,49–56], goats (n = 11) [34,36,38,43,51,52,55,57–60], sheep (n = 8) [34,38,43,51,52,55,56,58], dogs (n = 2) [38,54], pigs (n = 2) [36,61], donkeys (n = 1) [38], chicken (n = 1) [36], fish (n = 1) [36], and camels (n = 1) [36]. Seropositivity estimates varied widely in cattle (range: 0.8% to 85.9%), sheep (0.0% to 26.1%) and goats (2.6% to 46.9%). A summary of the study characteristics is presented in S1 Table. One study estimated the incidence of leptospirosis in both seroconverted sheep and goats to be 1.8 cases per 100 animal-months at risk each [34]. The meta-analysis performed on results from journal articles revealed an overall seroprevalence of bovine leptospirosis of 28.6% (95% confidence intervals [CI]: 4.7–76.5) by the MAT test and 26.6% (95% CI: 12.8–47.1) by ELISA. The overall seroprevalence in 3,311 cattle included was 28.2% (95% CI: 12.0–53.0), and high between-study heterogeneity was found (*I*^2^ = 96.7%, τ^2^ = 1.4) (Fig 4). Similarly, the pooled seroprevalence of caprine leptospirosis by MAT that included 1,850 goats was 11.0% (95% CI: 5.4–21.2), with a high between-study heterogeneity being observed (*I*^2^ = 78.8%, τ^2^ = 0.4) (Fig 4).

Wildlife were investigated with a specific focus on rodents and small mammals (n = 4) [24,31,35,38], gazelles (n = 2) [60,62], elands (n = 2) [60,62], giraffes (n = 2) [60,62], topis (n = 2) [54,60], rhinos (n = 2) [54,63], wildebeests (n = 1) [39], hartebeests (kongonis, n = 1) [64], dik-diks (n = 1) [64], bongos (n = 1) [64], cheetahs (n = 1) [57], lions (n = 1) [62], waterbucks (n = 1) [64], zebras (n = 1) [58], hippopotamuses (n = 1) [58], sunis (n = 1) [58], buffaloes (n = 1) [39], elephants (n = 1) [62], and impala (n = 1) [64]. A summary of seropositive animals and other study characteristics is given in S1 Table.

Several studies on human populations were found in healthcare [25,27,28,30,33,37,41,42,65] and community [20,26,32,38,52,65] settings. Seropositivity estimates in community populations ranged from 2.5% to 25.7% and those in hospitals from 0.5% to 25.0% (S1 Table). Estimates of the burden of disease in Kenya from the Leptospirosis Epidemiology Reference Group (LERG) of the World Health Organisation (WHO) revealed the disease is responsible for a mean estimate of 67,596 DALYs annually in the country [66]. The LERG group also estimated an annual incidence of human leptospirosis of 2.9 cases per 100,000 population in the country [67].

### Serovar and species distribution

**Fig 5:**
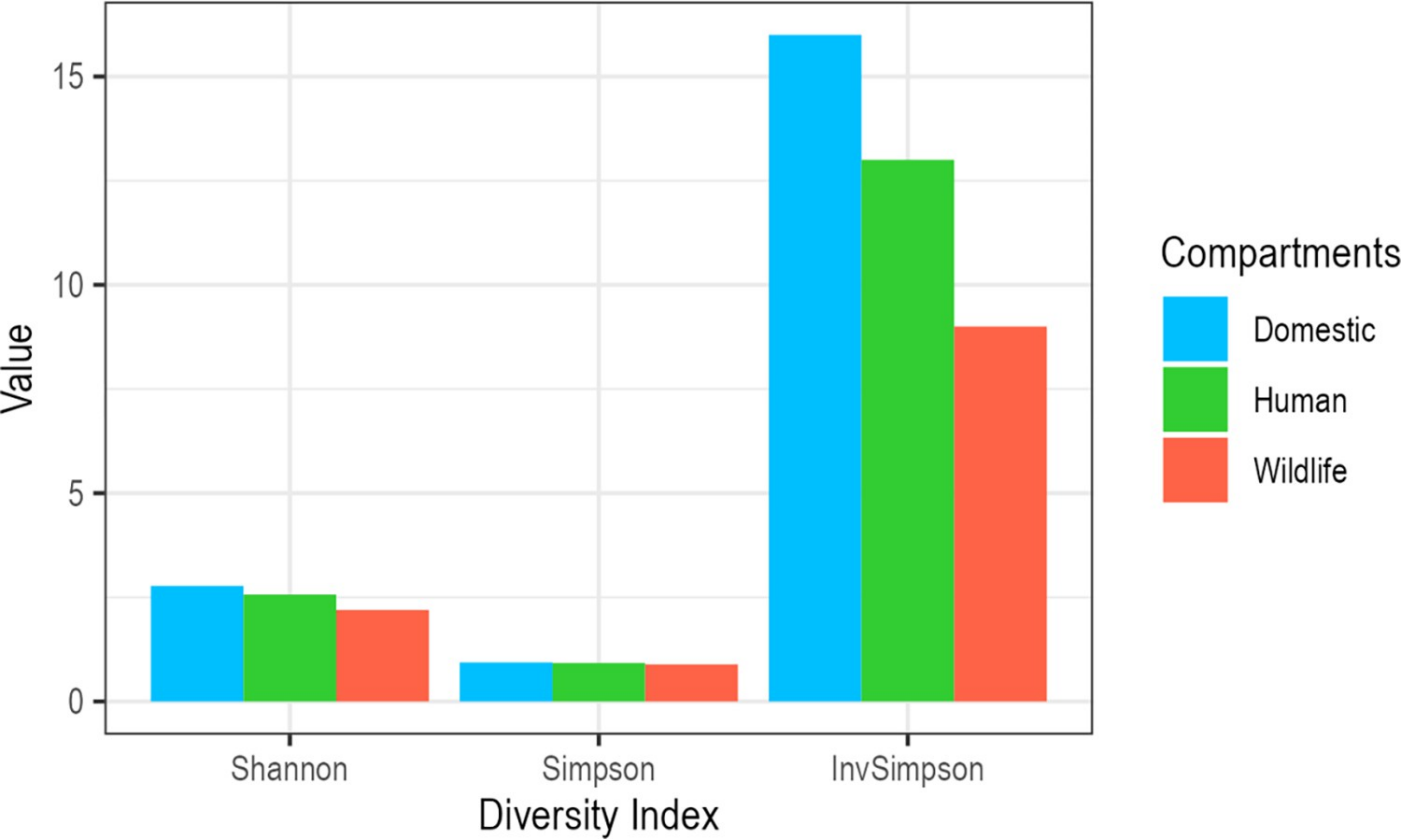
Serogroup diversity as determined by MAT testing on various hosts classified into three epidemiological compartments. The diversity indexes demonstrate serogroup richness and evenness. Kruskal-Wallis test results showed no statistically significant differences in the serogroup diversity across the domestic, wildlife and human hosts using all three indexes (p-values = 0.37).

The MAT panel used in studies varied substantially (Fig 3). The five most frequently investigated serogroups were Icterohaemorrhagiae (n = 28), Sejroe (n = 26), Grippotyphosa (n = 22), Canicola (n = 20), and Australis (n = 19). The least comprised Mini (n = 5), Panama (n = 2), Shermani (n = 2), Djasiman (n = 1) and Manhao (n = 1). When the diversity of serogroups observed via serology in hosts categorised into human, wildlife and domestic animal compartments was determined, a greater serogroup diversity was observed in domestic animals using all diversity indexes applied (Shannon = 2.77, Simpson = 0.94, Inverse Simpson = 16). The human compartment showed less diversity (Shannon = 2.56, Simpson = 0.92, Inverse Simpson = 13) and wildlife species showed the least serogroup diversity (Shannon = 2.2, Simpson = 0.89, Inverse Simpson = 9) (Fig 5). The three separate Kruskal-Wallis tests conducted to assess the serogroup diversity estimates in the three compartments for each of the three indexes (Shannon, Simpson, and Inverse Simpson) all returned identical results, with a p-value of 0.37.

Three studies used molecular tools to identify genomospecies, with single locus sequence typing (SLST) based on the *secY* gene showing *L*. *interrogans* [24,35,36] and *L*. *kirschneri* [35].

### Clinical signs and symptoms

Three dated studies documented the clinical presentation of human leptospirosis from culture-positive patients [41, 42, 68]. Common clinical presentations in increasing order were jaundice (5.9%), hepatomegaly (23.5%), meningism (23.5%), muscle tenderness (35.3%), respiratory tract manifestations (41.2%), rigour (41.2%), conjunctival injection (47.1%), digestive tract manifestations (58.8%), myalgia (58.8%), backache (64.7%), excess urobilin in urine (70.0%), joint pain (82.4%), headache (88.2%), abnormal urine deposits (90%), and albuminuria (90%).

### Risk factors

Risk factors for exposure to leptospires both serologically and using PCR were either mentioned as likely risk factors due to higher positivity rates or estimated using univariable and multivariable models in 15 studies. In cattle, high seropositivity or odds ratios were observed in older animals [52], both males and females [22,52], grazing in communal sites [22], being near wildlife [22,23], and in larger herds [23,50,59]. Cattle, sheep and goats from areas with high rainfall amounts had high leptospiral seroprevalence [49]. Mature pigs and those kept with other species were also shown to have higher odds of seropositivity [61]. Mice were shown to have a higher occurrence of leptospires than rats by PCR [24,35]. Rodents from environments more conducive to harbouring leptospires, possibly due to factors such as reduced elevation, flooding risk, and proximity to rubbish dumps and drainages, had higher odds of testing positive by PCR in Nairobi’s Kibera informal settlement [35]. Lastly, migratory wildlife and free-ranging cattle were more likely to be seropositive than non-migratory and captive wild animals [39]. Seropositivity in humans was high in occupationally exposed slaughterhouse workers [20], pastoral farmers [26], and sugarcane workers [41,42,65].

### Outbreaks and pathology studies

Outbreak investigations of animal leptospirosis were reported in cattle, pigs, sheep and goats [40,43,44,53] and an associated post-mortem investigation of cattle, sheep, goats, and pigs from the affected farms, experimental and natural infections was also found [69]. The main findings were nephritis with inconstant jaundice. A post-mortem investigation of canine leptospirosis not associated with any reported outbreak was found and was the earliest report of the disease in Kenya [70]. Icteric appearance of tissues, nephritis, hepatomegaly, splenomegaly, and enteritis were found in the infected dogs.

Human leptospirosis has been associated with outbreaks of acute febrile illnesses in pastoral communities in the former North-eastern province [25,71] and in patients in Malindi [72]. The largest known outbreak reported in humans was in Bungoma in the western region (referred to as “swamp fever” at the time) that caused several hospitalisations of school-going children and adults from surrounding communities, affecting more than 100 students and causing more than 30 casualties [73–77].

### Leptospirosis awareness

Three studies on the knowledge, attitudes and practices surrounding leptospirosis were found, assessing the level of awareness in communities. These were one journal article and two PhD theses. An investigation in Nakuru revealed that only 18 out of 66 (27.3%) animal health service providers interviewed considered leptospirosis to be associated with bovine abortions, indicating low awareness [78]. An assessment in Nairobi found that only 3.8% of clinical practitioners and laboratory personnel had suspected leptospirosis in their clinical practice. Additionally, only 2.3% would consider leptospirosis in the differential diagnosis of acute febrile illnesses of unknown origin. None of the health facilities surveyed in Nairobi (15/15, 100%) had the diagnostic capacity for MAT testing and rapid diagnostic tests (RDTs) [79]. Lastly, half (11/22, 50%) of pastoralist respondents interviewed in Laikipia and Maasai Mara (16/29, 55.2%) considered leptospirosis as a zoonotic disease that is not necessarily tick-transmitted, along with anthrax, brucellosis, helminthiases, trypanosomiasis and diarrhoeal diseases [80].

## Discussion

We present the first in-depth systematic review of human and animal leptospirosis in Kenya encompassing literature from 1951 to 2022. There was a spike in the publication numbers in the period 2010 to 2020. Most studies reported on the central, coastal, southern and western regions of the country. The vast arid and semiarid lands that are home to large populations of Kenya’s indigenous livestock kept in pastoral and agropastoral systems [81] and important wildlife ecosystems [82] were underrepresented. Studies mostly utilised serological methods to investigate antibodies against various serovars, leaving the genetic diversity of leptospires in Kenya largely unexplored. There is little awareness of leptospirosis among farmers, animal health and medical practitioners. Differing MAT panels and cut-offs were used leading to possible heterogenous results for the country, with the most common serogroup investigated being Icterohaemorrhagiae. Serovar diversity between humans, wildlife and domestic species did not significantly vary, suggesting similar environmental exposure across these epidemiological compartments and the need for more studies studying the transmission dynamics. The widespread presence of leptospires in various domestic animals, wildlife, and humans in various settings demonstrates the need for raising awareness in communities and among healthcare practitioners, and investments in diagnostics for more effective disease control and prevention.

A regional bias was observed, with most reported investigations situated in the central, coastal, southern, and western regions of Kenya, a probable leptospirosis risk profile of the country. The semi-intensive farming systems and relatively higher precipitation make these areas conducive transmission zones for leptospirosis. Urban centres such as Nairobi in the south-central region of the country have a burgeoning urban population and complex human-domestic-wildlife interfaces that facilitate bacterial transmission. However, large livestock populations exist in the arid and semi-arid eastern and northern regions of Kenya, along with communal grazing fields and the presence of rodents and wildlife which can all facilitate leptospiral transmission. *Leptospira* spp. were also implicated in outbreaks of acute febrile illnesses in human populations in these areas [25,71], justifying increased focus on these regions.

Several studies on leptospirosis in Kenya are out of date and utilised serology and culture, with animal inoculation to isolate the organism for diagnosis. The changing epidemiological landscape brought about by dynamic disease drivers such as climate change, urbanisation, agricultural intensification, population growth and land use changes necessitate up-to-date studies. These should focus on risk factors, circulating serovars, local strain diversity, and the transmission dynamics via a One Health prism to enable policy for rolling out adequate diagnostics, disease prevention and control measures. The increased awareness of non-malarial fevers after 2010 when the WHO recommended parasitological confirmation of malaria before treatment [11] could have led to renewed interest in leptospirosis research as demonstrated by the spike in journal articles and grey literature numbers.

Our review revealed that the country currently lacks adequate capacity for MAT testing, given that the test was more popular in studies before the year 2000. The only three studies that applied MAT testing from the year 2000 onwards were done in collaboration with laboratories with established MAT testing in Tanzania and Germany [34,61,63]. The MAT test is useful in determining infective serogroups which are important in clinical diagnosis and to understand the local epidemiological transmission of the disease. Vaccine development also currently relies on this information as immunity is relatively serovar-specific and vaccines should include homologous or antigenically-similar serovars to be effective [83]. However, cross-reactions between serogroups are common, and initial high titres against a noninfective serovar followed by high titres against the truly infective serovar (termed paradoxical reactions) are documented [83] and have been observed in small ruminants in Kenya [34]. Determining infective serovars can, therefore, be improved with repeat sampling. A challenge also exists in determining important serogroups to comprise a MAT panel. The commonly investigated serogroups of Icterohaemorrhagiae, Sejroe, Grippotyphosa, Canicola, and Australis were the most prevalent. Both the RGA and Ictero No.1 strains were used in some studies to represent Icterohaemorrhagiae because of their serological difference arising from an additional thermolabile antigen in Ictero No. 1 [84], thereby slightly contributing to the over-representation of the serogroup in MAT panels. Less included serogroups such as Djasiman could lead to underreporting, especially since the serogroup has been implicated in renal dysfunction in neighbouring Uganda [85]. Therefore, even though MAT testing has been utilised in the country for several years, there is a need for MAT panels that are as inclusive as possible to update Kenya’s information on circulating serovars, the resources available in a study notwithstanding. Lastly, the use of various MAT cut-offs can lead to heterogeneity between studies, making it difficult to compare studies in the country. The use of local strains in MAT panels can increase diagnostic sensitivity and has been recommended for African settings [86]. The WOAH recommends the use of reference strains of serovars, available from the WOAH reference lab [15], MAT cut-off titres of 1:100 and diagnosis of acute infection in animals be made with a four-fold rise in antibody titres between acute and convalescent serum samples [87]. The last study to culture for leptospires in Kenya was in the late 1980s, indicating a considerable gap in research efforts focussing on culturing and isolating leptospires from biological or environmental samples. Culture of leptospires can be performed for routine propagation of laboratory strains, or from clinical and environmental samples such as blood, cerebrospinal fluid, urine, livers, kidneys, water and soil using the EMJH media [88]. ELISA has also been used in the country but has been shown to have low diagnostic accuracy in endemic regions when compared to MAT when determining seroprevalence in humans [89,90], and may not always distinguish between vaccinated and infected cattle [87].

There is a severe lack of molecular investigations of leptospires, which are best placed to determine *Leptospira* species and characterise the bacteria according to its current WGS-based taxonomy. The earliest of the eleven PCR-based investigations found was from 2011, demonstrating a relatively recent adoption of molecular diagnostics in leptospirosis research in Kenya. The *lipL32* target is commonly used to identify pathogenic leptospires (now sub-clade P1) [91]. However, the *rrs* gene is more sensitive in detecting leptospires in clinical samples due to the potential role of “intermediate” leptospires (now sub-clade P2) which lack the *lipl32* locus [92]. A high prevalence of leptospirosis caused by these intermediates has been demonstrated in febrile patients in Ecuador, indicating their understated role in clinical disease [93]. *Leptospira* detection via PCR is typically done using loci that are present in all bacteria such as *gyrB*, *rrs*, and *secY*, or those found in pathogenic leptospires only such as *lipL32*, *ligA*, *ligB*, and *flaB* with varying diagnostic accuracies being reported [92,94]. Strain diversity can be demonstrated using SLST, but multi-locus sequence typing (MLST) utilising housekeeping genes and the PubMLST database is robust in directly determining leptospiral genomospecies and illustrating local strain diversity. This approach has been fruitful in characterising novel strains in endemic countries [95]. Additional PCR methods such as viability PCR (vPCR) can identify live/intact leptospires from environmental samples [96], thus showing the added public health risk which can have application in disease surveillance in Kenya, especially when isolate recovery is not possible or successful. The utilisation of WGS on leptospiral strains offers unmatched resolution in determining the phylogenetic relatedness of isolates from various regions and determining pathogen characteristics such as sequence types, antimicrobial resistance genes, virulence factors, plasmid profiles, and serovars. Antimicrobial susceptibility testing of leptospiral isolates currently lacks official breakpoints for many antimicrobial agents, and the few studies performed show a lack of emergence of antimicrobial resistance for the bacteria [97].

Surveillance in diverse hosts is important in the country as most studies were focused on cattle, sheep and goats which are important reservoirs in the country. The pooled seroprevalence estimates found in cattle were similar to other endemic countries [98,99], and those in goats were similar to those from Iran but lower than other estimates from Africa [98,99]. The heterogeneity observed was substantial, a likely result of the various diagnostic cut-offs, as well as other varying study characteristics. Future meta-analyses with more statistical power could undertake sub-group and meta-regression analyses to identify and characterise potential causes of between-study heterogeneity such as population demographics, study designs and geographical locations. This will enable harmonisation of studies in the country which can improve the comparability of results. Additionally, despite conducting a risk of bias assessment, our meta-analytic estimates are limited by the quality of the included studies and may be influenced by selection and publication bias. However, we did not attempt publication bias analyses due to the high heterogeneity found. Despite this, these analyses reveal a high but previously understated presence of cattle and caprine leptospirosis in the country. Efforts to reduce the disease prevalence in animals and increase productivity are crucial. Furthermore, the absence of comprehensive surveillance systems means the economic burden and contribution of human leptospirosis to febrile illnesses, as well as kidney, liver, neurological, respiratory, maternal and neonatal diseases, remain inadequately understood and likely underestimated.

Less or no focus was placed on rodents and various wildlife such as bats which could be important reservoirs as indicated by regional studies [100]. The few studies on dogs, pigs, and donkeys in the country give an unclear picture of the role of these hosts on leptospiral epidemiology in Kenya. Our investigation on the diversity of serovars in humans, domestic and wildlife revealed higher diversity in domestic animals that did not statistically differ from humans and wildlife. While the diversity measures used are sensitive to sample sizes [101], results obtained suggest similar environmental exposure of these three epidemiological compartments and the need for veterinary public health and One Health interventions to minimise exposure. Further research on the transmission dynamics of leptospirosis at the human-domestic-wildlife interface is needed to refine control options.

The risk posed to food vendors, consumers and others in the food value chain is largely unexplored in the country. A PCR-positive beef meat sample was found in grey literature [36] prompting curiosity about the uncommon finding and its implications for food safety. A high seropositivity of freshwater fish has been demonstrated in neighbouring Tanzania [102]. The use of slaughterhouse surveillance in studying leptospiral epidemiology can be a cost-effective way of screening animal/food samples with large geographical reach [103]. Studies on environmental samples are also lacking despite environmental exposure through occupations such as sugarcane farming and fishing being important risk factors in the country. Investigation of leptospirosis, which is an environmentally-driven zoonosis, extends beyond humans and animals and is best understood using a One Health approach that acknowledges the inter-relatedness of human, animal and environmental health. There is also a lack of estimates on the burden of animal leptospirosis and its incidence in important animal hosts such as cattle, with only one prospective cohort study found and revealing exceedingly higher incidence rates of small ruminant leptospirosis when compared to estimates in humans [34]. New approaches to estimating animal disease burden from the Global Burden of Animal Diseases program can help generate the burden of animal leptospirosis in Kenya which can guide policy and resource allocation [104]. Environmental drivers of the disease and the seasonality of human leptospirosis in Kenya are also largely undescribed, with the little current data showing a lack of seasonality of small ruminant leptospirosis [34]. However, recent work revisiting the 2004 outbreak of human leptospirosis in Bungoma shows that most cases occurred during or soon after the long rains (also MAM rains–March, April, May) [105]. Most investigations in this review were carried out in areas with higher precipitation, which may represent a risk profile of the disease in the country. However, reported cases can be utilised to develop spatio-temporal risk maps of leptospirosis that can help prioritise areas and seasons of increased leptospiral transmission in the country. Such risk maps can also incorporate data on temperature and rainfall patterns, thereby elucidating how future climate scenarios may impact the prevalence and geographic range of the disease.

Awareness of leptospirosis in farmers, animal health and medical practitioners was low. Additionally, it was demonstrated that several health centres are ill-equipped to diagnose leptospirosis due to the lack of RDT kits and infrastructure for MAT testing. While MAT is the serological gold standard that is useful in proving infective serogroups and determining the course of disease by agglutinating titres, it requires maintaining a panel of reference strains, dark field microscopes and knowledge of how to determine positives, making the interpretation subjective. This lack of diagnostic capacity can lead to undetected leptospirosis cases which can perpetuate a lack of awareness among health practitioners. There is a need for a tiered laboratory system to enable diagnostics in local and peripheral laboratories in high-risk areas using RDTs and the establishment of centralised reference labs for confirmatory tests, validation of diagnostics and to enable the country to participate in regional prevention and control efforts [106].

The clinical presentation of leptospirosis was non-distinguishing from the few studies that described the clinical signs and symptoms. Common clinical presentations for acute illness reported globally include fever, chills, headache, and myalgia. Conjunctival suffusion as an ocular manifestation is regarded as a distinguishing sign of human leptospirosis [4] even though it has been observed in patients with other infections such as Hantavirus [107], thus requiring careful consideration of the local epidemiological picture. Knowledge of the clinical signs and symptoms could be useful in syndromic surveillance, and future studies should determine the positive predictive values of various clinical signs and symptoms to inform policy that enables early clinical suspicion from health practitioners [108].

We found no official reports of prevention or control plans for leptospirosis in the country, even though a strategic plan exists for the prevention and control of zoonotic diseases with a One Health approach [109]. Prevention can be achieved through the use of personal protective equipment to prevent occupational exposure, protecting water and food sources from contamination from animal reservoirs, increasing awareness in communities to enable timely diagnosis, and controlling rodent infestations by clearing breeding sites such as garbage pits and improving sanitation [110]. Vaccination is a viable option for the control of animal leptospirosis and recent advances in vaccinology such as mRNA vaccines could hold the key to a universal leptospiral vaccine protective against a broad spectrum of pathogenic serovars [111], a prospect that could benefit from Africa’s growing capacity for producing advanced vaccines [112].

## Conclusion

In conclusion, leptospirosis research spanning more than 70 years revealed a regional bias towards the central, coastal, southern and western parts of Kenya. Despite this, widespread exposure of leptospires was demonstrated in various hosts across the country. Moving forward, future research efforts should prioritise isolating and characterising the bacteria using molecular approaches. PCR-based tests offer a viable option for surveillance in humans, animals and the environment to characterise locally-circulating strains even when culturing for the bacteria is not possible or successful. This will enhance our understanding of the transmission dynamics in a One Health space. Additionally, investigating risk factors for exposure for various humans and animals, and paying attention to underrepresented reservoir hosts is essential for a more robust understanding of leptospiral epidemiology in Kenya. Efforts to implement rapid diagnostic tests for peripheral laboratories and MAT testing for central laboratories are vital for timely and accurate leptospirosis diagnosis. Lastly, raising awareness of leptospirosis among farmers, health practitioners and veterinary professionals is crucial for early recognition and diagnosis that can lead to effective management. Education initiatives targeting at-risk populations such as farmers and slaughterhouse workers can empower individuals with knowledge about risks, prevention and control strategies, thereby contributing to better public health outcomes.

## Supporting information

S1 Table A summary of the characteristics of publications included in the systematic review. (DOCX)

S1 Data Database search terms used to find literature on leading electronic databases. The PRISMA checklist is also presented.
(DOCX)

S2 Data A summary of the data extracted from the 66 included studies. (DOCX)
