# Supplementary material for: Epidemiology of human and animal leptospirosis in Kenya: A systematic review and meta-analysis of disease occurrence, serogroup diversity and risk factors
Source: PLoS Negl Trop Dis. 2024 Sep 27;18(9):e0012527. doi: 10.1371/journal.pntd.0012527 (PMC11463743; doi:10.1371/journal.pntd.0012527)
Supplement: S1 Table — (DOCX) [file pntd.0012527.s001.docx]

# **Supplementary Information:**

## **Epidemiology of human and animal leptospirosis in Kenya: A systematic review and meta-analysis of disease occurrence, serogroup diversity and risk factors**

**Martin Wainaina, Joseph Wasonga, Elizabeth Anne Jessie Cook**

Supplementary Table 1: A summary of the characteristics of publications included in the systematic review

| **Burden of disease and human incidence (LERG studies)** | **First Author** | **Study period** | **Study sites** | **Study description** | **Burden and incidence estimates** |
| --- | --- | --- | --- | --- | --- |
|  | Torgerson [1] | Unmentioned | Country-wide | Burden of disease human  (Hospital and community cases) | Mean estimate of 67,596 disability-adjusted life years (DALYs) annually |
|  | Costa [2] | Unmentioned | Country-wide | Burden of disease humans  (Hospital and community cases) | Annual incidence of 2.9 cases per 100,000 population estimated |
| **Cross-sectional studies** | **First Author** | **Study period** | **Study sites** | **Study description** | **Positivity and diagnostics** |
|  | D'souza [3] | 1980 to 1982 | Various | Cross-sectional animal (domestic) | Serology: MAT cut-off 1:200  Cattle (723/2864) |
|  | Gossler [4] | Unmentioned | Kabete | Cross-sectional animal (domestic) | Serology: MAT cut-off 1:400  Cattle (121/200) |
|  | Macharia [5] | Unmentioned | Nyandarua | Cross-sectional animal (domestic) | Serology: MAT cut-off 1:100  Cattle (161/326), Sheep and Goats (196/357) |
|  | Munyua [6] | 1991 to 1994 | Various | Cross-sectional animal (domestic) | Serology: Plate agglutination test cut-off 1:50  Goats (305/650) |
|  | Murray [7] | Unmentioned | Kikuyu | Cross-sectional animal (domestic) | Serology: MAT cut-off unmentioned, agglutination lysis test and/or agglutination test  Dog (9/165), Cattle (29/55), Topi (3/5), Cheetah (0/1), Rhino (0/1) |
|  | Nakeel [8] | July to September 2012 | Kajiado | Cross-sectional animal (domestic) | Serology: PrioCHECK® *L. hardjo* indirect ELISA  Cattle (54/248) |
|  | Ndarathi [9] | Unmentioned | Kajiado | Cross-sectional animal (domestic) | Serology: MAT cut-off 1:100  Cattle [*L. wolfii*] (113/618), Cattle [*L. hardjo*] (176/618), Cattle [*L. grippotyphosa*] (5/618), Sheep [*L. hardjo*] (2/100), Goats [*L. wolfii*] (4/151), Goats [*L. hardjo*] (5/151) |
|  | Ngugi [10] | May to July 2018 | Busia | Cross-sectional animal (domestic) | Serology: MAT cut-off 1:40  Pig (83/252) |
|  | Njaramba [11] | 2018 November | Nairobi | Cross-sectional animal (domestic) | PCR: *secY* target  Cattle [beef] (1/58), Goat meat (0/23), Pig [pork] (0/8), Camel meat (0/3), Chicken meat (0/4), Fish meat (0/8) |
|  | Nthiwa [12] | September 2016 to July 2017 | Masai Mara | Cross-sectional animal (domestic) | Serology: PrioCHECK® *L. hardjo* indirect ELISA  Cattle (275/1170) |
|  | Rajeev [13] | June to July 2012 | Laikipia | Cross-sectional animal (domestic) | Serology: Linnodee® *L. hardjo* ELISA  Cattle (138/391) |
|  | Wanyangu [14] | Unmentioned | Various | Cross-sectional animal (domestic) | Serology: MAT cut-off 1:100  Goats (145/898) |
|  | Wanyangu [15] | Unmentioned | Rusinga Island | Cross-sectional animal (domestic) | Serology: MAT cut-off 1:50  Cattle (4/208), Sheep (0/97), Goats (2/48) |
|  | Wanyangu [16] | Unmentioned | Various | Cross-sectional animal (domestic) | Serology: MAT cut-off 1:100  Sheep (84/322), Goats (55/434) |
|  | Wanyangu [17] | Unmentioned | Various | Cross-sectional animal (domestic and wildlife) | Serology: MAT cut-off 1:50  Wildebeest (71/178), Buffalo (15/127), Cattle (17/86)  Culture:  Wildebeest (0/62) |
|  | Ball [18] | 1962 to 1963 | Various | Cross-sectional animal human (community), domestic and wildlife | Serology: MAT cut-off 1:100  Rodents (12/547), Goats (11/92), Sheep (2/25), Donkey (2/3), Dog (0/5), Cattle (61/150)  Culture:  Humans (4/113) |
|  | Bett [19] | Unmentioned | Tana River and Garissa | Cross-sectional human (community) | Serology: Panbio® IgM ELISA  Humans (244/948) |
|  | Cook [20] | May 2011 and October 2012 | Busia | Cross-sectional human (community) | Serology: Panbio® IgM ELISA  Humans (99/737) |
|  | Macharia [21] | Unmentioned | Nyandarua and Turkana | Cross-sectional human (community) and animal (domestic) | Serology: MAT cut-off 1:200  Cattle (209/765), Sheep and Goats (136/726), Humans (17/681) |
|  | Forrester [22] ‡ | 1961 to 1967 | Various | Cross-sectional human (hospital and community) | Serology: MAT cut-off 1:100  Humans (107/944) |
|  | Krandendonk [23] ‡ | 1961 to 1967 | Results are reported by Forrester et al. | Cross-sectional human (hospital and community) | Results are reported by Forrester et al. |
|  | De Geus [24] † | 1967-1969 | Results are reported by De Geus et al. | Cross-sectional human (hospital) | Results are reported by De Geus et al. |
|  | De Geus [25] *,† | August to September 1967 | Results are reported by De Geus et al. | Cross-sectional human (hospital) | Results are reported by De Geus et al. |
|  | De Geus [26] *, † | September 1968 to February 1969 | Former Kwale district | Cross-sectional human (hospital) | Serology: MAT cut-off 1:3000 or tenfold rise in paired sera  Seroprevalence undetermined  Culture:  Humans (17/130) |
|  | De Geus [27] *, † | March to July 1969 | Former Nyanza province | Cross-sectional human (hospital) | Serology: MAT cut-off 1:3000 or tenfold rise in paired sera  Humans (2/281)  Culture:  Humans (7/281) |
|  | De Geus [28] * | 1971 | Results are reported by De Geus et al. | Cross-sectional human (hospital) | Results are reported by De Geus et al. |
|  | Manabe [29] | March 26 2018 to September 30 2019 | Various | Cross-sectional human (hospital) | PCR: *flab* and *lipL32* targets  Humans (0/158) |
|  | Masakhwe [30] | Unmentioned | Various | Cross-sectional human (hospital) | PCR: 16S rRNA (*rrs*) target  Prevalence undetermined (n=796) |
|  | Munira [31] | January 10 2011 to March 11 2011 | Various | Cross-sectional human (hospital) | Serology: Panbio® IgM ELISA  Humans (1/182) |
|  | Njoroge [32] | 2009 to 2010 | Garissa | Cross-sectional human (hospital) | PCR: Unmentioned PCR target  Humans (0/304) |
|  | Waggoner [33] | January 16, 2014 to July 3 2015 | Various | Cross-sectional human (hospital) | PCR: 16S rRNA (*rrs*) target  Humans (1/385) |
|  | Fischer-Tenhagen [34] | 1987 to 1997 | Various | Cross-sectional wildlife | Serology: MAT cut-off 1:50  Black rhino (2/49), White rhino (3/9) |
|  | Halliday [35] | September to October 2008 | Kibera | Cross-sectional wildlife | PCR: *secY* target  Rodents (41/224) |
|  | Liyai [36] | Unmentioned | Marigat | Cross-sectional wildlife | Amplicon sequencing: 16S rRNA (*rrs*) target  Rodents (<1% abundance, n=54) |
|  | Twigg [37] | Unmentioned | Various | Cross-sectional wildlife | Serology: MAT cut-off 1:30  Elephant (3/17), Giraffe (3/3), Lion (1/3), Eland (1/2), Thomson's gazelle (1/1) |
|  | Wainaina [38] | December 2013 to March 2014 | Tana River and Garissa | Cross-sectional wildlife | Serology: MyBioSource® Rat IgG ELISA  Rodents (14/56)  PCR: *secY* target  Rodents (28/67) |
|  | Wanyangu [39] | Unmentioned | Various | Cross-sectional wildlife | Serology: MAT cut-off 1:50  Bongo (0/1), Dik-dik (0/6), Eland (1/8), Giraffe (7/42), Grant's gazelle (0/8), Thomson's gazelle (0/4), Hippopotamus (1/1), Impala (1/22), Kongoni (0/15), Suni (0/6), Topi (0/1), Waterbuck (1/33), Burchell's Zebra (0/2), Grevy's Zebra (2/3) |
| **Diagnostic test evaluations** | **First Author** | **Study period** | **Study sites** | **Study description** | **Positivity** |
|  | Liu [40] | 2008 to 2014 | Unmentioned | Diagnostic test evaluation human (community) | PCR: *lipL32* target  Humans (0/15) |
|  | Terpstra [41] | Unmentioned | Various | Diagnostic test evaluation human (hospital and community) | Serology: MAT cut-off unmentioned, ELISA  Positivity undetermined (n=690) |
|  | Wolff [42] | May 1961 | Unmentioned | Diagnostic test evaluation human (community) | Serology: MAT cut-off 1:100  Positivity undetermined (n=153) |
|  | Grolla [43] | December 2006 | Malindi | Diagnostic test evaluation human (hospital) | PCR: 16S rRNA (*rrs*) target  Humans (1/33) |
| **Isolate characterisation studies** | **First Author** | **Study period** | **Study sites** | **Study description** | **Isolate** |
|  | Dikken [44] | 1967 to 1968 | Various | Isolate characterisation study wildlife (rodent) | Culture and serology: CAAT  Novel serovar kanana (novel reference strain Kanana, serogroup Tarassovi);  Novel serovar lambwe (novel reference strain Lambwe, serogroup Autumnalis); Novel serovar kenya (novel reference strain Njenga, serogroup Ballum) |
|  | Dikken [45] | 1968 | Former Kwale district | Isolate characterisation study human (hospital) | Culture and serology: CAAT  Novel serovar kwale (novel reference strain Julu, serogroup Pyrogenes) |
|  | Dikken [46] | 1968 | Ramisi | Isolate characterisation study human (hospital) | Culture and serology: CAAT  Novel serovar ramisi (novel reference strain Musa, serogroup Australis) |
|  | Dikken [47] | 1971 | Kisumu | Isolate characterisation study human (hospital) | Culture and serology: CAAT  Novel serovar nyanza (novel reference strain Kibos, serogroup Hebdomadis) |
| **Knowledge, Attitudes and Practices (KAP) studies** | **First Author** | **Study period** | **Study sites** | **Study description** | **Findings** |
|  | Ndeereh [48] | Unmentioned | Masai Mara and Laikipia | KAP study human (community) | Only half of pastoral farmers regarded leptospirosis as a zoonosis that is not necessarily tick-borne (11/22). |
|  | Okumu [49] | 2010 | Nakuru | KAP study human (community) | Few animal health practitioners associated leptospirosis with bovine abortions (18/56). |
|  | Orodi [50] | August to December 2019 | Nairobi | KAP study human (community) | Few healthcare practitioners and laboratory personnel suspected leptospirosis in clinical practice (5/133). Inadequate capacity for MAT testing in healthcare facilities in the country’s capital city, Nairobi, was determined (15/15). |
| **Longitudinal Studies** | **First Author** | **Study period** | **Study sites** | **Study description** | **Positivity and Incidence estimates** |
|  | Wainaina [51] | September 2014 to June 2015 | Tana River | Longitudinal/prospective cohort animal (domestic) | Serology: MAT cut-off 1:100  Sheep (4/86), Goats (44/227)  Serological incidence rates:  Sheep (0.018 cases per animal-months at risk), Goats (0.018 cases per animal-months at risk)  PCR: *lipL32* target  Sheep and Goats (0/313) |
| **Outbreak investigations** | **First Author** | **Study period** | **Study sites** | **Study description** | **Positivity** |
|  | Burdin [52] | 1956 | Various | Outbreak animal (domestic) | Serology: MAT cut-off 1:100  Cattle (186/1802), Cattle, Sheep and Goats (12/80) |
|  | Mulei [53] | Unmentioned | Unmentioned | Outbreak animal (domestic) | Serology: MAT cut-off titre unmentioned  Cattle (6/9) |
|  | Tabel [54] | 1975 | Kikuyu | Outbreak animal (domestic) | Culture: MAT cut-off 1:100  Cattle (49/57)  Culture:  Serovar Grippotyphosa isolated |
|  | Burdin [55] | May 1956 | Nanyuki | Outbreak animal (cattle) | Positivity undetermined, diagnostics unmentioned |
|  | Wurapa [56] | December 15 to December 18 | Various | Outbreak human (hospital and community) | PCR: PCR target unmentioned  Positives unmentioned (n=21) |
|  | Ari [57] | July 18, 2005 | Garissa | Outbreak human (hospital) | Serology: Panbio® IgM ELISA  Humans (3/12) |
|  | ProMED mail [58-62] | May 2004 | Bungoma | Outbreak humans (community) | In May 2004, the first ProMED alert signalled 26 deaths from leptospirosis. By mid-July, new cases persisted, particularly in four primary schools, with over 100 pupils hospitalized in Webuye district hospital. The outbreak was originally reported at Chesamisi Secondary School in Kimilili, where over 100 students were hospitalized and six fatalities were communicated. Additionally, two pupils from nearby primary schools and five villagers succumbed to the disease. |
|  | Woods [63] | December 1997 | Garissa | Outbreak humans (community) | Diagnostics unmentioned, positivity undetermined.  Patients who met the case definition for haemorrhagic fever, but were negative for Rift Valley fever virus were also positive for leptospires. |
| **Post-mortem (pathology) investigations** | **First Author** | **Study period** | **Study sites** | **Study description** | **Hosts** |
|  | Burdin [64] | Unmentioned | Unmentioned | Pathology study animal (domestic) | Cattle, Sheep, Goats, Pigs |
|  | Piercy [65] | 1951 | Kabete | Pathology study animal (domestic) | Dog |
| **Study protocol for future investigation** | **First Author** | **Study period** | **Study sites** | **Study description** | **Proposed samples** |
|  | Gachohi [66] | Unmentioned | Kibera | Study protocol animal (domestic) and human (community) | Humans, pigs and or dogs, soil, water |

CAAT: Cross agglutination absorption test, ELISA: Enzyme-linked immunosorbent assay, LERG: Leptospirosis Epidemiology Reference Group, MAT: Microscopic agglutination test, PCR: Polymerase chain reaction.

* Studies reporting on the same population/samples

† Thesis reporting on published studies but included due to extra information not available in constituent papers

‡ Studies reporting on the same population/samples

**References**

1. Torgerson PR, Hagan JE, Costa F, Calcagno J, Kane M, Martinez-Silveira MS, et al. Global burden of leptospirosis: Estimated in terms of disability adjusted life years. PLoS Negl Trop Dis. 2015;9(10):e0004122. Epub 2015/10/03. doi: 10.1371/journal.pntd.0004122. PubMed PMID: 26431366.

2. Costa F, Hagan JE, Calcagno J, Kane M, Torgerson P, Martinez-Silveira MS, et al. Global morbidity and mortality of leptospirosis: A systematic review. PLoS Negl Trop Dis. 2015;9(9):e0003898. Epub 20150917. doi: 10.1371/journal.pntd.0003898. PubMed PMID: 26379143; PubMed Central PMCID: PMCPMC4574773.

3. D'souza CF. Occurrence of bovine leptospirosis in Kenya. Kenya: University of Nairobi; 1983.

4. Gössler R, Hünermund G. [Serological studies on cattle in catchment area of Kabete (Kenia). 2. Determination of antibodies against *Mycobacterium paratuberculosis*, *Brucella*, *Salmonella*, *Pasteurella multocida*, *Listeria* and *Leptospira*]. Berl Munch Tierarztl Wochenschr. 1973;86(14):267-70. PubMed PMID: 4199742.

5. Macharia S, Mulei CM, Gathuma J, Kagiko M. Serological survey of leptospiral antibodies in cattle, sheep and goats in Nyandarua district of Kenya. Bull Anim Health Prod Afr. 1994;42:335-7.

6. Munyua SM. Reproductive performance and wastage in Goats in arid and semi arid areas of Kenya with special emphasis on pre-weaning mortality. Kenya: University of Nairobi; 1997.

7. Murray M. Animal diseases of East Africa: A study of their incidence and pathology [D.V.M.]. Scotland: University of Glasgow (United Kingdom); 1969.

8. Nakeel M, Arimi S, Kitala P, Nduhiu G, Njenga J, Wabacha J. A sero-epidemiological survey of brucellosis, Q-fever and leptospirosis in livestock and humans and associated risk factors in kajiado county-Kenya. J Trop Dis. 2016;4(3):8. doi: 10.4172/2329-891X.1000215.

9. Ndarathi CM, D'Souza C, Waghela S. The prevalence of leptospirosis in Maasai livestock in Kenya. Bull Anim Health Prod Afr. 1991;39(4):419-21. PubMed PMID: CABI:19922266194.

10. Ngugi JN, Fèvre EM, Mgode GF, Obonyo M, Mhamphi GG, Otieno CA, et al. Seroprevalence and associated risk factors of leptospirosis in slaughter pigs; A neglected public health risk, western Kenya. BMC Vet Res. 2019;15(1):403. Epub 20191108. doi: 10.1186/s12917-019-2159-3. PubMed PMID: 31703588; PubMed Central PMCID: PMCPMC6842184.

11. Njaramba J. Molecular identification of vertebrate sources and potential zoonotic pathogens in the meat value chain from selected vendors in Nairobi, Kenya. Kenya: University of Nairobi; 2020.

12. Nthiwa D, Alonso S, Odongo D, Kenya E, Bett B. Zoonotic pathogen seroprevalence in cattle in a wildlife-livestock interface, Kenya. EcoHealth. 2019;16(4):712-25. Epub 20191114. doi: 10.1007/s10393-019-01453-z. PubMed PMID: 31728795; PubMed Central PMCID: PMCPMC6910896.

13. Rajeev M, Mutinda M, Ezenwa VO. Pathogen exposure in cattle at the livestock-wildlife interface. EcoHealth. 2017;14(3):542-51. Epub 20170503. doi: 10.1007/s10393-017-1242-0. PubMed PMID: WOS:000414153000010.

14. Wanyangu SW, Angolio A, Wamwayi HM. Further serological evidence for caprine leptospirosis in Kenya. East Afr Agr Forest J. 1993;59(2):137-43. PubMed PMID: CABI:19952208408.

15. Wanyangu S, Waitkins S, D'Souza C, Mbogo S. Leptospirosis in Kenya: low serological prevalence in cattle, sheep and goats on Rusinga Island in Kenya. Bull Anim Health Prod Afr. 1988;36(2):188-9.

16. Wanyangu SW, Angolio A, Macharia S, Litamoi JK, Odongo OM. A preliminary serological survey for leptospiral agglutinins in sheep and goats of Kenya. East Afr Agr Forest J. 1990;56(1/4):15-9. PubMed PMID: CABI:19942209624.

17. Wanyangu SW, Olubayo RO, Rositter PB, Waitkins SA. The study of the ecology and prevalence of leptospirosis in large wild ruminants and domesticated bovines found in Kenya. Isr J Vet Med. 1987;43(4):340-1. PubMed PMID: BIOSIS:PREV198834105663.

18. Ball MG. Animal hosts of leptospires in Kenya and Uganda. Am J Trop Med Hyg. 1966;15(4):523-30. doi: 10.4269/ajtmh.1966.15.523. PubMed PMID: 4957422.

19. Bett B, Said MY, Sang R, Bukachi S, Wanyoike S, Kifugo SC, et al. Effects of flood irrigation on the risk of selected zoonotic pathogens in an arid and semi-arid area in the eastern Kenya. PLoS One. 2017;12(5):e0172626. Epub 20170531. doi: 10.1371/journal.pone.0172626. PubMed PMID: 28562600; PubMed Central PMCID: PMCPMC5450996.

20. Cook EA, de Glanville WA, Thomas LF, Kariuki S, Bronsvoort BM, Fèvre EM. Risk factors for leptospirosis seropositivity in slaughterhouse workers in western Kenya. Occup Environ Med. 2017;74(5):357-65. Epub 2016/12/04. doi: 10.1136/oemed-2016-103895. PubMed PMID: 27913579; PubMed Central PMCID: PMCPMC5520261.

21. Macharia SM. A comparative sero-epidemiological survey for the prevalence of *Leptospira* antibodies in domestic animals and man in Nyandarua and Turkana districts of Kenya. Kenya: University of Nairobi; 1989.

22. Forrester AT, Kranendonk O, Turner LH, Wolff JW, Bohlander HJ. Serological evidence of human leptospirosis in Kenya. East Afr Med J. 1969;46(9):497-506. Epub 1969/09/01. PubMed PMID: 5363301.

23. Kranendonk O, Forrester ATT, Turner LH, Wolff JW, de Rijk-Bohlander HJ. Leptospirosis bij mensen en knaagdieren in Kenia. Ned Tijdschr Geneeskd. 1971.

24. De Geus A. Human leptospirosis in rural Kenya: University of Amsterdam; 1971.

25. De Geus A, Kranendonk O, Bohlander HJ. Clinical leptospirosis in Kwale District, Coast Province, Kenya. East Afr Med J. 1969;46(9):491-6. Epub 1969/09/01. PubMed PMID: 5363300.

26. De Geus A, Wolff JW, Timmer VE. Clinical leptospirosis in Kenya (1): A clinical study in Kwale District, Coast Province. East Afr Med J. 1977;54(3):115-24. Epub 1977/03/01. PubMed PMID: 885095.

27. De Geus A, Wolff JW, Timmer VE. Clinical leptospirosis in Kenya (II): A field study in Nyanza Province. East Afr Med J. 1977;54(3):125-32. Epub 1977/03/01. PubMed PMID: 885096.

28. De Geus A. [Acute leptospirosis in humans in Kenya]. Ned Tijdschr Geneeskd. 1971;115(4):178-80. PubMed PMID: 5100430.

29. Manabe YC, Betz J, Jackson O, Asoala V, Bazan I, Blair PW, et al. Clinical evaluation of the BioFire Global Fever Panel for the identification of malaria, leptospirosis, chikungunya, and dengue from whole blood: a prospective, multicentre, cross-sectional diagnostic accuracy study. Lancet Infect Dis. 2022;22(9):1356-64. Epub 20220615. doi: 10.1016/S1473-3099(22)00290-0. PubMed PMID: 35716700; PubMed Central PMCID: PMCPMC9420791.

30. Masakhwe C, Ochanda H, Nyakoe N, Ochiel D, Waitumbi J. Frequency of Epstein - Barr virus in patients presenting with acute febrile illness in Kenya. PLoS One. 2016;11(5):e0155308. Epub 20160510. doi: 10.1371/journal.pone.0155308. PubMed PMID: 27163791; PubMed Central PMCID: PMCPMC4862666.

31. Munira AS. Documentation of the exposure of Kenyan residents to zoonotic diseases. Kenya: University of Nairobi; 2012.

32. Njoroge RN, Wurapa EK, Waitumbi JN, Breiman RF, Kariuki Njenga M. The etiology of acute febrile illness in patients presenting to Garissa Provincial Hospital in Northeastern Province, Kenya. Am J Trop Med Hyg. 2011;85:136.

33. Waggoner J, Brichard J, Mutuku F, Ndenga B, Heath CJ, Mohamed-Hadley A, et al. Malaria and Chikungunya detected using molecular diagnostics among febrile Kenyan children. Open Forum Infect Dis. 2017;4(3):ofx110. Epub 20170529. doi: 10.1093/OFID/OFX110. PubMed PMID: 28702473; PubMed Central PMCID: PMCPMC5505337.

34. Fischer-Tenhagen C, Hamblin C, Quandt S, Frolich K. Serosurvey for selected infectious disease agents in free-ranging black and white rhinoceros in Africa. J Wildl Dis. 2000;36(2):316-23. doi: 10.7589/0090-3558-36.2.316. PubMed PMID: WOS:000086854000016.

35. Halliday JEB, Knobel DL, Allan KJ, Bronsvoort BMdC, Handel I, Agwanda B, et al. Urban leptospirosis in Africa: A cross-sectional survey of *Leptospira* infection in rodents in the Kibera urban settlement, Nairobi, Kenya. Am J Trop Med Hyg. 2013;89(6):1095-102. Epub 20130930. doi: 10.4269/ajtmh.13-0415. PubMed PMID: WOS:000328726100009; PubMed Central PMCID: PMCPMC3854886.

36. Liyai R, Kimita G, Masakhwe C, Abuom D, Mutai B, Onyango DM, et al. The spleen bacteriome of wild rodents and shrews from Marigat, Baringo County, Kenya. PeerJ. 2021;9:e12067. Epub 2021/09/25. doi: 10.7717/peerj.12067. PubMed PMID: 34557350; PubMed Central PMCID: PMCPMC8418798.

37. Twigg GI, Sies SK, Hughes DM. Evidence of leptospirosis in some large East African mammals. Afr J Ecol. 1970;8(1):197-8. doi: 10.1111/j.1365-2028.1970.tb00840.x.

38. Wainaina M, Bett B, Ontiri E, Picozzi K, Agwanda B, Strand T, et al. *Leptospira* bacteria detected in rodents in Tana River and Garissa counties of Kenya. Infect Ecol Epidemiol. 2018;8(1). doi: 10.1080/20008686.2018.1547093.

39. Wanyangu SW, Rossitter PB, Olubayo RO, Wafula JS, Waitkins SA. Leptospiral agglutinins in the wild ungulates found in Kenya. Trop Vet. 1989;7(3-4):185-9. PubMed PMID: BIOSIS:PREV199191005733.

40. Liu J, Ochieng C, Wiersma S, Ströher U, Towner JS, Whitmer S, et al. Development of a TaqMan array card for acute-febrile-illness outbreak investigation and surveillance of emerging pathogens, including ebola virus. J Clin Microbiol. 2016;54(1):49-58. Epub 20151021. doi: 10.1128/JCM.02257-15. PubMed PMID: 26491176; PubMed Central PMCID: PMCPMC4702733.

41. Terpstra WJ, Njenga R, Korver H, Ligthart GS. ELISA for the detection of leptospirosis in Kenya. East Afr Med J. 1987;64(1):49-54.

42. Wolff JW, Bohlander HJ. Evaluation of Galton's macroscopic slide test for the serodiagnosis of leptospirosis in human serum samples. Ann Soc Belges Med Trop Parasitol Mycol. 1966;46((1)):123-32. PubMed PMID: BIOSIS:PREV19674800123198.

43. Grolla A, Mehedi M, Lindsay R, Bosio C, Duse A, Feldmann H. Enhanced detection of Rift Valley fever virus using molecular assays on whole blood samples. J Clin Virol. 2012;54(4):313-7. Epub 20120524. doi: 10.1016/j.jcv.2012.04.022. PubMed PMID: 22632901; PubMed Central PMCID: PMCPMC3398164.

44. Dikken H, Timmer VE, Njenga R. Three new leptospiral serovars from Kenya. Trop Geogr Med. 1981;33(4):343-6. PubMed PMID: 7342381.

45. Dikken H, Kmety E, de Geus A, Timmer VE. A new leptospiral serovar in the Pyrogenes serogroup. Trop Geogr Med. 1979;31(3):405-8. PubMed PMID: 524450.

46. Dikken H, Kmety E, De Geus A, Timmer VE. A new leptospiral serovar in the Australis serogroup. Trop Geogr Med. 1979;31(2):263-8. PubMed PMID: 505557.

47. Dikken H, Kmety E, de Geus A, Adinarayanan N, Timmer VE. Two new *Leptospira* serovars belonging to the Hebdomadis serogroup. Trop Geogr Med. 1978;30(4):537-42. PubMed PMID: 749290.

48. Ndeereh DR. Molecular epidemiology of spotted fever group rickettsioses and Q fever at the wildlife-livestock interface in Maasai Mara and Laikipia ecosystems, Kenya. Kenya: University of Nairobi; 2016.

49. Okumu TA. Infectious abortion and associated risk factors in dairy cattle farms in Nakuru district, Kenya: University of Nairobi; 2014.

50. Orodi NI, Gachohi J, Wanjihia V. Leptospirosis diagnostic capacity in public health facilities within Nairobi County, Kenya. TIJPH. 2022;10(1):231-45. doi: 10.21522/TIJPH.2013.10.01.Art019. PubMed PMID: CABI:20220205340.

51. Wainaina M, Lindahl JF, Dohoo I, Mayer-Scholl A, Roesel K, Mbotha D, et al. Longitudinal study of selected bacterial zoonoses in small ruminants in Tana River County, Kenya. Microorganisms. 2022;10(8). Epub 2022/08/27. doi: 10.3390/microorganisms10081546. PubMed PMID: 36013964; PubMed Central PMCID: PMCPMC9414833.

52. Burdin M, Froyd G, Ashford W. Leptospirosis in Kenya due to *Leptospira grippotyphosa*. Vet Rec. 1958;70:830–5. PubMed Central PMCID: PMCitem type: Journal article.

53. Mule C, Macharia S, Mbuthia P. An outbreak of bovine leptospirosis due to *Leptospira hardjo* and *Leptospira pomona* in a zero-grazing dairy herd in Kenya. Bull Anim Health Prod Afr. 1994;42:327-8.

54. Tabel H, Losos G. Report on an outbreak of bovine leptospirosis in Kenya due to *Leptospira grippotyphosa*. Bull Anim Health Prod Afr. 1979;27(1):61–4.

55. Burdin ML, Froyd G. Bovine leptospirosis in Kenya. Nature. 1957;179(4570):1140-. doi: 10.1038/1791140a0. PubMed PMID: 13430814.

56. Wurapa EK, Kambi J, Lumbaso S, Oluoch D, Abdirizak M, Batonjo G. Investigation of a suspected outbreak of acute febrile illness in Malindi, Kenya in December 2010. Am J Trop Med Hyg. 2011;85(6):387.

57. Ari MD, Guracha A, Fadeel MA, Njuguna C, Njenga MK, Kalani R, et al. Challenges of establishing the correct diagnosis of outbreaks of acute febrile illnesses in Africa: The case of a likely *Brucella* outbreak among nomadic pastoralists, Northeast Kenya, March-July 2005. Am J Trop Med Hyg. 2011;85(5):909-12. doi: 10.4269/ajtmh.2011.11-0030. PubMed PMID: 22049048; PubMed Central PMCID: PMCPMC3205640.

58. ProMED-mail. PRO/AH/EDR> Leptospirosis - Kenya (Western Districts) (04) Archive Number: 20040713.1879 2004 [cited 2023 14.02.2023].

59. ProMED-mail. PRO/AH/EDR> Leptospirosis - Kenya (western districts) (03) Archive Number: 20040707.1818 2004 [cited 2023 14.02.2023].

60. ProMED-mail. PRO/EDR> Leptospirosis - Kenya (western districts) (02) Archive Number: 20040619.1634 2004 [cited 2023 14.02.2023].

61. ProMED-mail. Leptospirosis - Kenya (western districts) Archive Number: 20040617.1616 2004 [cited 2023 14.02.2023].

62. ProMED-mail. PRO/EDR> Leptospirosis - Kenya (Bungoma): RFI Archive Number: 20040616.1613 2004 [cited 2023 14.02.2023].

63. Woods CW, Karpati AM, Grein T, McCarthy N, Gaturuku P, Muchiri E, et al. An outbreak of Rift Valley fever in northeastern Kenya, 1997-98. Emerg Infect Dis. 2002;8(2):138.

64. Burdin ML. Renal histopathology of leptospirosis caused by *Leptospira grippotyphosa* in farm animals in Kenya. Res Vet Sci. 1963;4(3):423-31. doi: 10.1016/S0034-5288(18)34854-9.

65. Piercy SE. Canine leptospirosis in Kenya. Vet Rec. 1951;63(25):425-6. doi: 10.1136/vr.63.25.425. PubMed PMID: 14855948.

66. Gachohi J, Karanja S, Wanyoike S, Gitahi N, Bukachi S, Ngure K. Sourcing eco-epidemiological field parameters to describe a slum household-based pathogenic *Leptospira* population dynamics simulation model in Kenya: A pseudo-longitudinal study protocol. Vet Scie Res Review. 2016;2(3):66-75. doi: 10.17582/journal.vsrr/2016.2.3.66.75.
