# Supplementary material for: Epidemiology of human and animal leptospirosis in Kenya: A systematic review and meta-analysis of disease occurrence, serogroup diversity and risk factors
Source: PLoS Negl Trop Dis. 2024 Sep 27;18(9):e0012527. doi: 10.1371/journal.pntd.0012527 (PMC11463743; doi:10.1371/journal.pntd.0012527)
Supplement: S2 Data — (DOCX) [file pntd.0012527.s003.docx]

# **Supplementary Information:**

## **Epidemiology of human and animal leptospirosis in Kenya: A systematic review and meta-analysis of disease occurrence, serogroup diversity and risk factors**

**Martin Wainaina, Joseph Wasonga, Elizabeth Anne Jessie Cook**

| **Article ID** | **Title** | **Authors** | **Year** | **Article Type** | **Decade** | **Sampling Period** | **Study Type** | **Study Design** | **Population** |
| --- | --- | --- | --- | --- | --- | --- | --- | --- | --- |
| [1] | A comparative sero-epidemiological survey for the prevalence of *Leptospira* antibodies in domestic animals and man in Nyandarua and Turkana districts of Kenya | Macharia SM | 1989 | Thesis | 1980-1989 | unmentioned | Domestic and human | Cross-sectional | community |
| [2] | A preliminary serological survey for leptospiral agglutinins in sheep and goats of Kenya | Wanyangu SW et al | 1990 | Journal article | 1990-1999 | unmentioned | Domestic | Cross-sectional | not applicable |
| [3] | Animal hosts of leptospires in Kenya and Uganda | Ball MG | 1966 | Journal article | 1960-1969 | 1962 to 1963 | Domestic and human | Cross-sectional | community |
| [4] | Bovine leptospirosis in Kenya | Burdin ML,Froyd G | 1957 | Journal article | 1950-1959 | May 1956 | Domestic | Outbreak | not applicable |
| [5] | Challenges of establishing the correct diagnosis of outbreaks of acute febrile illnesses in Africa: The case of a likely *Brucella* outbreak among nomadic pastoralists, Northeast Kenya, March-July 2005 | Ari MD et al | 2011 | Journal article | 2010-2019 | July 18, 2005 | Human | Outbreak | hospital |
| [6] | Clinical evaluation of the BioFire Global Fever Panel for the identification of malaria, leptospirosis, chikungunya, and dengue from whole blood: a prospective, multicentre, cross-sectional diagnostic accuracy study | Manabe YC et al | 2022 | Journal article | 2020-2022 | March 26, 2018, to Sept 30, 2019 | Human | Cross-sectional | hospital |
| [7] | Clinical leptospirosis in Kenya (1): a clinical study in Kwale District, Cost Province | De Geus A,Wolff JW,Timmer VE | 1977 | Journal article | 1970-1979 | September 1968 to February 1969 | Human | Cross-sectional | hospital |
| [8] | Clinical leptospirosis in Kenya (II): A field study in Nyanza Province | De Geus A,Wolff JW,Timmer VE | 1977 | Journal article | 1970-1979 | March to July 1969 | Human | Cross-sectional | hospital |
| [9] | Clinical leptospirosis in Kwale District, Coast Province, Kenya | De Geus A,Kranendonk O,Bohlander HJ | 1969 | Journal article | 1960-1969 | August to September 1967 | Human | Cross-sectional | hospital |
| [10] | Development of a TaqMan Array Card for Acute-Febrile-Illness Outbreak Investigation and Surveillance of Emerging Pathogens, Including Ebola Virus | Liu J et al | 2016 | Journal article | 2010-2019 | 2008 to 2014 | Human | Diagnostic test evaluation | community |
| [11] | Documentation of the exposure of Kenyan residents to zoonotic diseases | Munira AS | 2012 | Thesis | 2010-2019 | January 10 2011 to March 11 2011 | Human | Cross-sectional | hospital |
| [12] | Effects of flood irrigation on the risk of selected zoonotic pathogens in an arid and semi-arid area in the eastern Kenya | Bett B et al | 2017 | Journal article | 2010-2019 | unmentioned | Human | Cross-sectional | community |
| [13] | ELISA for the detection of leptospirosis in Kenya | Terpstra WJ et al | 1987 | Journal article | 1980-1989 | unmentioned | Human | Diagnostic test evaluation | community |
| [14] | Evaluation of Galton's macroscopic slide test for the serodiagnosis of leptospirosis in human serum samples | Wolff JW,Bohlander HJ | 1966 | Journal article | 1960-1969 | May 1961 | Human | Diagnostic test evaluation | community |
| [15] | Frequency of Epstein - Barr Virus in Patients Presenting with Acute Febrile Illness in Kenya | Masakhwe C et al | 2016 | Journal article | 2010-2019 | unmentioned | Human | Cross-sectional | hospital |
| [16] | Further serological evidence or caprine leptospirosis in Kenya | Wanyangu SW,Angolio A,Wamwayi HM | 1993 | Journal article | 1990-1999 | unmentioned | Domestic | Cross-sectional | not applicable |
| [17] | Global Morbidity and Mortality of Leptospirosis: A Systematic Review | Costa F et al | 2015 | Journal article | 2010-2019 | unmentioned | Human | Burden of disease | hospital and community |
| [18] | Infectious abortion and associated risk factors in dairy cattle farms in Nakuru district, Kenya | Okumu TA | 2014 | Thesis | 2010-2019 | 2010 | Human | KAP study | community |
| [19] | Investigation of a suspected outbreak of acute febrile illness in Malindi, Kenya in December 2010 | Wurapa EK et al | 2011 | Abstract | 2010-2019 | Dec 15 to Dec 18 | Human | Outbreak | hospital and community |
| [20] | *Leptospira* bacteria detected in rodents in Tana River and Garissa counties of Kenya | Wainaina M et al | 2018 | Journal article | 2010-2019 | December 2013 to March 2014 | Wildlife | Cross-sectional | not applicable |
| [21] | Leptospiral agglutinins in the wild ungulates found in Kenya | Wanyangu SW et al | 1989 | Journal article | 1980-1989 | unmentioned | Wildlife | Cross-sectional | not applicable |
| [22] | Leptospirosis - Kenya (western districts) Archive Number: 20040617.1616 | ProMED-mail | 2004 | Outbreak alert | 2000-2009 | May 2004 | Human | Outbreak | community |
| [23] | Leptospirosis diagnostic capacity in public health facilities within Nairobi County, Kenya | Orodi NI,Gachohi J,Wanjihia V | 2022 | Journal article | 2020-2022 | August to December 2019 | Human | KAP study | community |
| [24] | Leptospirosis in Kenya: low serological prevalence in cattle, sheep and goats on Rusinga Island in Kenya | Wanyangu SW et al | 1988 | Journal article | 1980-1989 | unmentioned | Domestic | Cross-sectional | not applicable |
| [25] | Longitudinal Study of Selected Bacterial Zoonoses in Small Ruminants in Tana River County, Kenya | Wainaina M et al | 2022 | Journal article | 2020-2022 | September 2014 to June 2015 | Domestic | Longitudinal | not applicable |
| [26] | Malaria and Chikungunya Detected Using Molecular Diagnostics Among Febrile Kenyan Children | Waggoner J et al | 2017 | Journal article | 2010-2019 | January 16, 2014 and July 3, 2015 | Human | Cross-sectional | hospital |
| [27] | Molecular epidemiology of spotted fever group rickettsioses and Q fever at the wildlife-livestock interface in Maasai Mara and Laikipia ecosystems, Kenya | Ndeereh DR | 2016 | Thesis | 2010-2019 | unmentioned | Human | KAP study | community |
| [28] | Molecular identification of vertebrate sources and potential zoonotic pathogens in the meat value chain from selected vendors in Nairobi, Kenya | Njaramba J | 2020 | Thesis | 2020-2022 | 2018 November | Domestic | Cross-sectional | not applicable |
| [29] | Occurrence of bovine leptospirosis in Kenya | D'souza CF | 1983 | Thesis | 1980-1989 | 1980 to 1982 | Domestic | Cross-sectional | not applicable |
| [30] | Pathogen Exposure in Cattle at the Livestock-Wildlife Interface | Rajeev M,Mutinda M,Ezenwa VO | 2017 | Journal article | 2010-2019 | June to July 2012 | Domestic | Cross-sectional | not applicable |
| [31] | PRO/AH/EDR\> Leptospirosis - Kenya (western districts) (03) Archive Number: 20040707.1818 | ProMED-mail | 2004 | Outbreak alert | 2000-2009 | May 2004 | Human | Outbreak | community |
| [32] | PRO/AH/EDR\> Leptospirosis - Kenya (Western Districts) (04) Archive Number: 20040713.1879 | ProMED-mail | 2004 | Outbreak alert | 2000-2009 | May 2004 | Human | Outbreak | community |
| [33] | PRO/EDR\> Leptospirosis - Kenya (Bungoma): RFI Archive Number: 20040616.1613 | ProMED-mail | 2004 | Outbreak alert | 2000-2009 | May 2004 | Human | Outbreak | community |
| [34] | PRO/EDR\> Leptospirosis - Kenya (western districts) (02) Archive Number: 20040619.1634 | ProMED-mail | 2004 | Outbreak alert | 2000-2009 | May 2004 | Human | Outbreak | community |
| [35] | Reproductive performance and wastage in goats in arid and semi arid areas of Kenya with special emphasis on pre-weaning mortality | Munyua SJ | 1997 | Thesis | 1990-1999 | 1991 to 1994 | Domestic | Cross-sectional | not applicable |
| [36] | Risk factors for leptospirosis seropositivity in slaughterhouse workers in western Kenya | Cook EA et al | 2017 | Journal article | 2010-2019 | May 2011 and October 2012 | Human | Cross-sectional | community |
| [37] | Serological evidence of human leptospirosis in Kenya | Forrester AT et al | 1969 | Journal article | 1960-1969 | 1961 to 1967 | Human | Cross-sectional | hospital and community |
| [38] | Serological survey of leptospiral antibodies in cattle, sheep and goats in Nyandarua district of Kenya | Macharia SM et al | 1994 | Journal article | 1990-1999 | unmentioned | Domestic | Cross-sectional | not applicable |
| [39] | Seroprevalence and associated risk factors of leptospirosis in slaughter pigs; A neglected public health risk, western Kenya | Ngugi JN et al | 2019 | Journal article | 2010-2019 | May to July 2018 | Domestic | Cross-sectional | not applicable |
| [40] | Serosurvey for selected infectious disease agents in free-ranging black and white rhinoceros in Africa | Fischer-Tenhagen C et al | 2000 | Journal article | 2000-2009 | 1987 to 1997 | Wildlife | Cross-sectional | not applicable |
| [41] | Sourcing eco-epidemiological field parameters to describe a slum household-based pathogenic *Leptospira* population dynamics simulation model in Kenya: A pseudo-longitudinal study protocol | Gachohi J et al | 2016 | Journal article | 2010-2019 | not applicable | Domestic and human | Study protocol | community |
| [42] | The etiology of acute febrile illness in patients presenting to Garissa Provincial Hospital in Northeastern Province, Kenya | Njoroge RN et al | 2011 | Abstract | 2010-2019 | 2009 to 2010 | Human | Cross-sectional | hospital |
| [43] | The prevalence of leptospirosis in Maasai livestock in Kenya | Ndarathi CM,D'Souza C,Waghela S | 1991 | Journal article | 1990-1999 | unmentioned | Domestic | Cross-sectional | not applicable |
| [44] | The spleen bacteriome of wild rodents and shrews from Marigat, Baringo County, Kenya | Liyai R et al | 2021 | Journal article | 2020-2022 | unmentioned | Wildlife | Cross-sectional | not applicable |
| [45] | The study of the ecology and prevalence of leptospirosis in large wild ruminants and domesticated bovines found in Kenya | Wanyangu SW et al | 1987 | Abstract | 1980-1989 | unmentioned | Domestic and wildlife | Cross-sectional | not applicable |
| [46] | Urban leptospirosis in Africa: A cross-sectional survey of *Leptospira* infection in rodents in the Kibera urban settlement, Nairobi, Kenya | Halliday JE et al | 2013 | Journal article | 2010-2019 | September to October 2008 | Wildlife | Cross-sectional | not applicable |
| [47] | Zoonotic Pathogen Seroprevalence in Cattle in a Wildlife-Livestock Interface, Kenya | Nthiwa D et al | 2019 | Journal article | 2010-2019 | September 2016 to July 2017 | Domestic | Cross-sectional | not applicable |
| [48] | A sero-epidemiological survey of brucellosis, Q-fever and leptospirosis in livestock and humans and associated risk factors in Kajiado county-Kenya | Nakeel, MJ et al | 2016 | Journal article | 2010-2019 | July to September 2012 | Domestic | Cross-sectional | not applicable |
| [49] | Three new leptospiral serovars in Kenya | Dikken et al | 1981 | Journal article | 1980-1989 | 1967 to 1968 | Domestic | Isolate characterisation | not applicable |
| [50] | A new leptospiral serovar in the Pyrogenes serogroup | Dikken et al | 1979 | Journal article | 1970-1979 | 1968 | Human | Isolate characterisation | hospital |
| [51] | Renal histopathology of leptospirosis caused by *Leptospira* *grippotyphosa* in farm animals in Kenya | Burdin ML | 1963 | Journal article | 1960-1969 | unmentioned | Domestic | Pathology study | not applicable |
| [52] | An outbreak of bovine leptospirosis due to *Leptospira* *hardjo* and *Leptospira* *pomona* in a zero-grazing dairy herd in Kenya. | Mule CM, Macharia SM, Mbuthia PG | 1994 | Journal article | 1990-1999 | unmentioned | Domestic | Outbreak | not applicable |
| [53] | [Serological studies on cattle in catchment area of Kabete (Kenya). 2. Determination of antibodies against *Mycobacterium* *paratuberculosis*, *Brucella*, *Salmonella*, *Pasteurella* *multocida*, *Listeria* and *Leptospira*] | Gossler R,Hunermund G | 1973 | Journal article | 1970-1979 | unmentioned | Domestic | Cross-sectional | not applicable |
| [54] | Report of an outbreak of bovine leptospirosis in Kenya due to *Leptospira* Grippotyphosa | Tabel H, Losos G | 1979 | Journal article | 1970-1979 | 1975 | Domestic | Outbreak | not applicable |
| [55] | Animal Diseases of East Africa: A Study of Their Incidence and Pathology | Murray M | 1969 | Thesis | 1960-1969 | unmentioned | Domestic | Cross-sectional | not applicable |
| [56] | Acute leptospirose bij de mens in Kenia | Geus A | 1971 | Journal article | 1970-1979 | 1971 | Human | Cross-sectional | hospital |
| [57] | Leptospirosis bij mensen en knaagdieren in Kenia | Kranendonk et al | 1971 | Journal article | 1970-1979 | 1971 | Human | Cross-sectional | hospital |
| [58] | Human leptospirosis in rural Kenya | de Geus A | 1971 | Thesis | 1970-1979 | 1971 | Human | Cross-sectional | hospital |
| [59] | Evidence of leptospirosis on large East African mammals | Twigg et al | 1970 | Journal article | 1970-1979 | unmentioned | Wildlife | Cross-sectional | not applicable |
| [60] | A new leptospiral serovar in the Australis serogroup. | Dikken et al | 1979 | Journal article | 1970-1979 | 1968 | Human | Isolate characterisation | hospital |
| [61] | Two new *Leptospira* serovars belonging to the Hebdomadis serogroup | Dikken et al | 1978 | Journal article | 1970-1979 | 1971 | Human | Isolate characterisation | hospital |
| [62] | Leptospirosis in Kenya due to *Leptospira* *grippotyphosa* | Burdin ML,Froyd G,Ashford WA | 1958 | Journal article | 1950-1959 | 1956 | Domestic | Outbreak | not applicable |
| [63] | Canine leptospirosis in Kenya | Piercy et al | 1951 | Journal article | 1950-1959 | 1951 | Domestic | Pathology study | not applicable |
| [64] | Enhanced detection of Rift Valley fever virus using molecular assays on whole blood samples | Grolla et al | 2012 | Journal article | 2010-2019 | December 2006 | Human | Diagnostic test evaluation | hospital |
| [65] | Global Burden of Leptospirosis: Estimated in terms of Disability Adjusted Life Years. | Torgerson et al | 2015 | Journal article | 2010-2019 | unmentioned | Human | Burden of disease | hospital and community |
| [66] | An outbreak of Rift Valley fever in northeastern Kenya, 1997-98 | Woods et al | 2002 | Journal article | 2000-2009 | December 1997 | Human | Outbreak | community |

**References**

1. Macharia SM. A comparative sero-epidemiological survey for the prevalence of *Leptospira* antibodies in domestic animals and man in Nyandarua and Turkana districts of Kenya. Kenya: University of Nairobi; 1989.

2. Wanyangu SW, Angolio A, Macharia S, Litamoi JK, Odongo OM. A preliminary serological survey for leptospiral agglutinins in sheep and goats of Kenya. East Afr Agr Forest J. 1990;56(1/4):15-9. PubMed PMID: CABI:19942209624.

3. Ball MG. Animal hosts of leptospires in Kenya and Uganda. Am J Trop Med Hyg. 1966;15(4):523-30. doi: 10.4269/ajtmh.1966.15.523. PubMed PMID: 4957422.

4. Burdin ML, Froyd G. Bovine leptospirosis in Kenya. Nature. 1957;179(4570):1140-. doi: 10.1038/1791140a0. PubMed PMID: 13430814.

5. Ari MD, Guracha A, Fadeel MA, Njuguna C, Njenga MK, Kalani R, et al. Challenges of establishing the correct diagnosis of outbreaks of acute febrile illnesses in Africa: The case of a likely *Brucella* outbreak among nomadic pastoralists, Northeast Kenya, March-July 2005. Am J Trop Med Hyg. 2011;85(5):909-12. doi: 10.4269/ajtmh.2011.11-0030. PubMed PMID: 22049048; PubMed Central PMCID: PMCPMC3205640.

6. Manabe YC, Betz J, Jackson O, Asoala V, Bazan I, Blair PW, et al. Clinical evaluation of the BioFire Global Fever Panel for the identification of malaria, leptospirosis, chikungunya, and dengue from whole blood: a prospective, multicentre, cross-sectional diagnostic accuracy study. Lancet Infect Dis. 2022;22(9):1356-64. Epub 20220615. doi: 10.1016/S1473-3099(22)00290-0. PubMed PMID: 35716700; PubMed Central PMCID: PMCPMC9420791.

7. De Geus A, Wolff JW, Timmer VE. Clinical leptospirosis in Kenya (1): A clinical study in Kwale District, Coast Province. East Afr Med J. 1977;54(3):115-24. Epub 1977/03/01. PubMed PMID: 885095.

8. De Geus A, Wolff JW, Timmer VE. Clinical leptospirosis in Kenya (II): A field study in Nyanza Province. East Afr Med J. 1977;54(3):125-32. Epub 1977/03/01. PubMed PMID: 885096.

9. De Geus A, Kranendonk O, Bohlander HJ. Clinical leptospirosis in Kwale District, Coast Province, Kenya. East Afr Med J. 1969;46(9):491-6. Epub 1969/09/01. PubMed PMID: 5363300.

10. Liu J, Ochieng C, Wiersma S, Ströher U, Towner JS, Whitmer S, et al. Development of a TaqMan array card for acute-febrile-illness outbreak investigation and surveillance of emerging pathogens, including ebola virus. J Clin Microbiol. 2016;54(1):49-58. Epub 20151021. doi: 10.1128/JCM.02257-15. PubMed PMID: 26491176; PubMed Central PMCID: PMCPMC4702733.

11. Munira AS. Documentation of the exposure of Kenyan residents to zoonotic diseases. Kenya: University of Nairobi; 2012.

12. Bett B, Said MY, Sang R, Bukachi S, Wanyoike S, Kifugo SC, et al. Effects of flood irrigation on the risk of selected zoonotic pathogens in an arid and semi-arid area in the eastern Kenya. PLoS One. 2017;12(5):e0172626. Epub 20170531. doi: 10.1371/journal.pone.0172626. PubMed PMID: 28562600; PubMed Central PMCID: PMCPMC5450996.

13. Terpstra WJ, Njenga R, Korver H, Ligthart GS. ELISA for the detection of leptospirosis in Kenya. East Afr Med J. 1987;64(1):49-54.

14. Wolff JW, Bohlander HJ. Evaluation of Galton's macroscopic slide test for the serodiagnosis of leptospirosis in human serum samples. Ann Soc Belges Med Trop Parasitol Mycol. 1966;46((1)):123-32. PubMed PMID: BIOSIS:PREV19674800123198.

15. Masakhwe C, Ochanda H, Nyakoe N, Ochiel D, Waitumbi J. Frequency of Epstein - Barr virus in patients presenting with acute febrile illness in Kenya. PLoS One. 2016;11(5):e0155308. Epub 20160510. doi: 10.1371/journal.pone.0155308. PubMed PMID: 27163791; PubMed Central PMCID: PMCPMC4862666.

16. Wanyangu SW, Angolio A, Wamwayi HM. Further serological evidence for caprine leptospirosis in Kenya. East Afr Agr Forest J. 1993;59(2):137-43. PubMed PMID: CABI:19952208408.

17. Costa F, Hagan JE, Calcagno J, Kane M, Torgerson P, Martinez-Silveira MS, et al. Global morbidity and mortality of leptospirosis: A systematic review. PLoS Negl Trop Dis. 2015;9(9):e0003898. Epub 20150917. doi: 10.1371/journal.pntd.0003898. PubMed PMID: 26379143; PubMed Central PMCID: PMCPMC4574773.

18. Okumu TA. Infectious abortion and associated risk factors in dairy cattle farms in Nakuru district, Kenya: University of Nairobi; 2014.

19. Wurapa EK, Kambi J, Lumbaso S, Oluoch D, Abdirizak M, Batonjo G. Investigation of a suspected outbreak of acute febrile illness in Malindi, Kenya in December 2010. Am J Trop Med Hyg. 2011;85(6):387.

20. Wainaina M, Bett B, Ontiri E, Picozzi K, Agwanda B, Strand T, et al. *Leptospira* bacteria detected in rodents in Tana River and Garissa counties of Kenya. Infect Ecol Epidemiol. 2018;8(1). doi: 10.1080/20008686.2018.1547093.

21. Wanyangu SW, Rossitter PB, Olubayo RO, Wafula JS, Waitkins SA. Leptospiral agglutinins in the wild ungulates found in Kenya. Trop Vet. 1989;7(3-4):185-9. PubMed PMID: BIOSIS:PREV199191005733.

22. ProMED-mail. Leptospirosis - Kenya (western districts) Archive Number: 20040617.1616 2004 [cited 2023 14.02.2023].

23. Orodi NI, Gachohi J, Wanjihia V. Leptospirosis diagnostic capacity in public health facilities within Nairobi County, Kenya. TIJPH. 2022;10(1):231-45. doi: 10.21522/TIJPH.2013.10.01.Art019. PubMed PMID: CABI:20220205340.

24. Wanyangu S, Waitkins S, D'Souza C, Mbogo S. Leptospirosis in Kenya: low serological prevalence in cattle, sheep and goats on Rusinga Island in Kenya. Bull Anim Health Prod Afr. 1988;36(2):188-9.

25. Wainaina M, Lindahl JF, Dohoo I, Mayer-Scholl A, Roesel K, Mbotha D, et al. Longitudinal study of selected bacterial zoonoses in small ruminants in Tana River County, Kenya. Microorganisms. 2022;10(8). Epub 2022/08/27. doi: 10.3390/microorganisms10081546. PubMed PMID: 36013964; PubMed Central PMCID: PMCPMC9414833.

26. Waggoner J, Brichard J, Mutuku F, Ndenga B, Heath CJ, Mohamed-Hadley A, et al. Malaria and Chikungunya detected using molecular diagnostics among febrile Kenyan children. Open Forum Infect Dis. 2017;4(3):ofx110. Epub 20170529. doi: 10.1093/OFID/OFX110. PubMed PMID: 28702473; PubMed Central PMCID: PMCPMC5505337.

27. Ndeereh DR. Molecular epidemiology of spotted fever group rickettsioses and Q fever at the wildlife-livestock interface in Maasai Mara and Laikipia ecosystems, Kenya. Kenya: University of Nairobi; 2016.

28. Njaramba J. Molecular identification of vertebrate sources and potential zoonotic pathogens in the meat value chain from selected vendors in Nairobi, Kenya. Kenya: University of Nairobi; 2020.

29. D'souza CF. Occurrence of bovine leptospirosis in Kenya. Kenya: University of Nairobi; 1983.

30. Rajeev M, Mutinda M, Ezenwa VO. Pathogen exposure in cattle at the livestock-wildlife interface. EcoHealth. 2017;14(3):542-51. Epub 20170503. doi: 10.1007/s10393-017-1242-0. PubMed PMID: WOS:000414153000010.

31. ProMED-mail. PRO/AH/EDR> Leptospirosis - Kenya (western districts) (03) Archive Number: 20040707.1818 2004 [cited 2023 14.02.2023].

32. ProMED-mail. PRO/AH/EDR> Leptospirosis - Kenya (Western Districts) (04) Archive Number: 20040713.1879 2004 [cited 2023 14.02.2023].

33. ProMED-mail. PRO/EDR> Leptospirosis - Kenya (Bungoma): RFI Archive Number: 20040616.1613 2004 [cited 2023 14.02.2023].

34. ProMED-mail. PRO/EDR> Leptospirosis - Kenya (western districts) (02) Archive Number: 20040619.1634 2004 [cited 2023 14.02.2023].

35. Munyua SM. Reproductive performance and wastage in Goats in arid and semi arid areas of Kenya with special emphasis on pre-weaning mortality. Kenya: University of Nairobi; 1997.

36. Cook EA, de Glanville WA, Thomas LF, Kariuki S, Bronsvoort BM, Fèvre EM. Risk factors for leptospirosis seropositivity in slaughterhouse workers in western Kenya. Occup Environ Med. 2017;74(5):357-65. Epub 2016/12/04. doi: 10.1136/oemed-2016-103895. PubMed PMID: 27913579; PubMed Central PMCID: PMCPMC5520261.

37. Forrester AT, Kranendonk O, Turner LH, Wolff JW, Bohlander HJ. Serological evidence of human leptospirosis in Kenya. East Afr Med J. 1969;46(9):497-506. Epub 1969/09/01. PubMed PMID: 5363301.

38. Macharia S, Mulei CM, Gathuma J, Kagiko M. Serological survey of leptospiral antibodies in cattle, sheep and goats in Nyandarua district of Kenya. Bull Anim Health Prod Afr. 1994;42:335-7.

39. Ngugi JN, Fèvre EM, Mgode GF, Obonyo M, Mhamphi GG, Otieno CA, et al. Seroprevalence and associated risk factors of leptospirosis in slaughter pigs; A neglected public health risk, western Kenya. BMC Vet Res. 2019;15(1):403. Epub 20191108. doi: 10.1186/s12917-019-2159-3. PubMed PMID: 31703588; PubMed Central PMCID: PMCPMC6842184.

40. Fischer-Tenhagen C, Hamblin C, Quandt S, Frolich K. Serosurvey for selected infectious disease agents in free-ranging black and white rhinoceros in Africa. J Wildl Dis. 2000;36(2):316-23. doi: 10.7589/0090-3558-36.2.316. PubMed PMID: WOS:000086854000016.

41. Gachohi J, Karanja S, Wanyoike S, Gitahi N, Bukachi S, Ngure K. Sourcing eco-epidemiological field parameters to describe a slum household-based pathogenic *Leptospira* population dynamics simulation model in Kenya: A pseudo-longitudinal study protocol. Vet Scie Res Review. 2016;2(3):66-75. doi: 10.17582/journal.vsrr/2016.2.3.66.75.

42. Njoroge RN, Wurapa EK, Waitumbi JN, Breiman RF, Kariuki Njenga M. The etiology of acute febrile illness in patients presenting to Garissa Provincial Hospital in Northeastern Province, Kenya. Am J Trop Med Hyg. 2011;85:136.

43. Ndarathi CM, D'Souza C, Waghela S. The prevalence of leptospirosis in Maasai livestock in Kenya. Bull Anim Health Prod Afr. 1991;39(4):419-21. PubMed PMID: CABI:19922266194.

44. Liyai R, Kimita G, Masakhwe C, Abuom D, Mutai B, Onyango DM, et al. The spleen bacteriome of wild rodents and shrews from Marigat, Baringo County, Kenya. PeerJ. 2021;9:e12067. Epub 2021/09/25. doi: 10.7717/peerj.12067. PubMed PMID: 34557350; PubMed Central PMCID: PMCPMC8418798.

45. Wanyangu SW, Olubayo RO, Rositter PB, Waitkins SA. The study of the ecology and prevalence of leptospirosis in large wild ruminants and domesticated bovines found in Kenya. Isr J Vet Med. 1987;43(4):340-1. PubMed PMID: BIOSIS:PREV198834105663.

46. Halliday JEB, Knobel DL, Allan KJ, Bronsvoort BMdC, Handel I, Agwanda B, et al. Urban leptospirosis in Africa: A cross-sectional survey of *Leptospira* infection in rodents in the Kibera urban settlement, Nairobi, Kenya. Am J Trop Med Hyg. 2013;89(6):1095-102. Epub 20130930. doi: 10.4269/ajtmh.13-0415. PubMed PMID: WOS:000328726100009; PubMed Central PMCID: PMCPMC3854886.

47. Nthiwa D, Alonso S, Odongo D, Kenya E, Bett B. Zoonotic pathogen seroprevalence in cattle in a wildlife-livestock interface, Kenya. EcoHealth. 2019;16(4):712-25. Epub 20191114. doi: 10.1007/s10393-019-01453-z. PubMed PMID: 31728795; PubMed Central PMCID: PMCPMC6910896.

48. Nakeel M, Arimi S, Kitala P, Nduhiu G, Njenga J, Wabacha J. A sero-epidemiological survey of brucellosis, Q-fever and leptospirosis in livestock and humans and associated risk factors in kajiado county-Kenya. J Trop Dis. 2016;4(3):8. doi: 10.4172/2329-891X.1000215.

49. Dikken H, Timmer VE, Njenga R. Three new leptospiral serovars from Kenya. Trop Geogr Med. 1981;33(4):343-6. PubMed PMID: 7342381.

50. Dikken H, Kmety E, de Geus A, Timmer VE. A new leptospiral serovar in the Pyrogenes serogroup. Trop Geogr Med. 1979;31(3):405-8. PubMed PMID: 524450.

51. Burdin ML. Renal histopathology of leptospirosis caused by *Leptospira grippotyphosa* in farm animals in Kenya. Res Vet Sci. 1963;4(3):423-31. doi: 10.1016/S0034-5288(18)34854-9.

52. Mule C, Macharia S, Mbuthia P. An outbreak of bovine leptospirosis due to *Leptospira hardjo* and *Leptospira pomona* in a zero-grazing dairy herd in Kenya. Bull Anim Health Prod Afr. 1994;42:327-8.

53. Gössler R, Hünermund G. [Serological studies on cattle in catchment area of Kabete (Kenia). 2. Determination of antibodies against *Mycobacterium paratuberculosis*, *Brucella*, *Salmonella*, *Pasteurella multocida*, *Listeria* and *Leptospira*]. Berl Munch Tierarztl Wochenschr. 1973;86(14):267-70. PubMed PMID: 4199742.

54. Tabel H, Losos G. Report on an outbreak of bovine leptospirosis in Kenya due to *Leptospira grippotyphosa*. Bull Anim Health Prod Afr. 1979;27(1):61–4.

55. Murray M. Animal diseases of East Africa: A study of their incidence and pathology [D.V.M.]. Scotland: University of Glasgow (United Kingdom); 1969.

56. De Geus A. [Acute leptospirosis in humans in Kenya]. Ned Tijdschr Geneeskd. 1971;115(4):178-80. PubMed PMID: 5100430.

57. Kranendonk O, Forrester ATT, Turner LH, Wolff JW, de Rijk-Bohlander HJ. Leptospirosis bij mensen en knaagdieren in Kenia. Ned Tijdschr Geneeskd. 1971.

58. De Geus A. Human leptospirosis in rural Kenya: University of Amsterdam; 1971.

59. Twigg GI, Sies SK, Hughes DM. Evidence of leptospirosis in some large East African mammals. Afr J Ecol. 1970;8(1):197-8. doi: 10.1111/j.1365-2028.1970.tb00840.x.

60. Dikken H, Kmety E, De Geus A, Timmer VE. A new leptospiral serovar in the Australis serogroup. Trop Geogr Med. 1979;31(2):263-8. PubMed PMID: 505557.

61. Dikken H, Kmety E, de Geus A, Adinarayanan N, Timmer VE. Two new *Leptospira* serovars belonging to the Hebdomadis serogroup. Trop Geogr Med. 1978;30(4):537-42. PubMed PMID: 749290.

62. Burdin M, Froyd G, Ashford W. Leptospirosis in Kenya due to *Leptospira grippotyphosa*. Vet Rec. 1958;70:830–5. PubMed Central PMCID: PMCitem type: Journal article.

63. Piercy SE. Canine leptospirosis in Kenya. Vet Rec. 1951;63(25):425-6. doi: 10.1136/vr.63.25.425. PubMed PMID: 14855948.

64. Grolla A, Mehedi M, Lindsay R, Bosio C, Duse A, Feldmann H. Enhanced detection of Rift Valley fever virus using molecular assays on whole blood samples. J Clin Virol. 2012;54(4):313-7. Epub 20120524. doi: 10.1016/j.jcv.2012.04.022. PubMed PMID: 22632901; PubMed Central PMCID: PMCPMC3398164.

65. Torgerson PR, Hagan JE, Costa F, Calcagno J, Kane M, Martinez-Silveira MS, et al. Global burden of leptospirosis: Estimated in terms of disability adjusted life years. PLoS Negl Trop Dis. 2015;9(10):e0004122. Epub 2015/10/03. doi: 10.1371/journal.pntd.0004122. PubMed PMID: 26431366.

66. Woods CW, Karpati AM, Grein T, McCarthy N, Gaturuku P, Muchiri E, et al. An outbreak of Rift Valley fever in northeastern Kenya, 1997-98. Emerg Infect Dis. 2002;8(2):138.
